# Extracellular vesicle PD-L1 dynamics predict durable response to immune-checkpoint inhibitors and survival in patients with non-small cell lung cancer

**DOI:** 10.1186/s13046-022-02379-1

**Published:** 2022-06-02

**Authors:** Diego de Miguel-Perez, Alessandro Russo, Oscar Arrieta, Murat Ak, Feliciano Barron, Muthukumar Gunasekaran, Priyadarshini Mamindla, Luis Lara-Mejia, Christine B. Peterson, Mehmet E. Er, Vishal Peddagangireddy, Francesco Buemi, Brandon Cooper, Paolo Manca, Rena G. Lapidus, Ru-Ching Hsia, Andres F. Cardona, Aung Naing, Sunjay Kaushal, Fred R. Hirsch, Philip C. Mack, Maria Jose Serrano, Vincenzo Adamo, Rivka R. Colen, Christian Rolfo

**Affiliations:** 1 Center for Thoracic Oncology, Tisch Cancer Institute, Icahn School of Medicine at Mount Sinai, New York, NY USA; 2 Marlene and Stewart Greenebaum Comprehensive Cancer Center, University of Maryland School of Medicine, Baltimore, MD USA; 3 Medical Oncology Unit, A.O. Papardo & Department of Human Pathology, University of Messina, Messina, Italy; 4 Thoracic Oncology Unit, Instituto Nacional de Cancerología (INCan), Mexico City, Mexico; 5 Department of Radiology, University of Pittsburgh, Pittsburgh, PA USA; 6 Hillman Cancer Center, University of Pittsburgh Medical Center, Pittsburgh, PA USA; 7 The University of Texas MD Anderson Cancer Center, Houston, TX USA; 8 Fondazione IRCCS Istituto Nazionale Dei Tumori Di Milano, Milan, Italy; 9 Luis Carlos Sarmiento Angulo Cancer Treatment and Research Center (CTIC) / Foundation for Clinical and Applied Cancer Research (FICMAC) / Molecular Oncology and Biology Systems Research Group (Fox-G), Universidad El Bosque, Bogotá, Colombia; 10 Departments of Investigational Cancer Therapeutics, The University of Texas MD Anderson Cancer Center, Houston, TX USA; 11 GENYO Centre for Genomics and Oncological Research, Pfizer/ University of Granada/ Andalusian Regional Government, PTS Granada, Granada, Spain

**Keywords:** Extracellular vesicles, PD-L1, Biomarkers, Immunotherapy, NSCLC

## Abstract

**Background:**

Immune-checkpoint inhibitors (ICIs) changed the therapeutic landscape of patients with lung cancer. However, only a subset of them derived clinical benefit and evidenced the need to identify reliable predictive biomarkers. Liquid biopsy is the non-invasive and repeatable analysis of biological material in body fluids and a promising tool for cancer biomarkers discovery. In particular, there is growing evidence that extracellular vesicles (EVs) play an important role in tumor progression and in tumor-immune interactions. Thus, we evaluated whether extracellular vesicle PD-L1 expression could be used as a biomarker for prediction of durable treatment response and survival in patients with non-small cell lung cancer (NSCLC) undergoing treatment with ICIs.

**Methods:**

Dynamic changes in EV PD-L1 were analyzed in plasma samples collected before and at 9 ± 1 weeks during treatment in a retrospective and a prospective independent cohorts of 33 and 39 patients, respectively.

**Results:**

As a result, an increase in EV PD-L1 was observed in non-responders in comparison to responders and was an independent biomarker for shorter progression-free survival and overall survival. To the contrary, tissue PD-L1 expression, the commonly used biomarker, was not predictive neither for durable response nor survival.

**Conclusion:**

These findings indicate that EV PD-L1 dynamics could be used to stratify patients with advanced NSCLC who would experience durable benefit from ICIs.

**Supplementary Information:**

The online version contains supplementary material available at 10.1186/s13046-022-02379-1.

## Background

Immune checkpoint inhibitors (ICIs) have revolutionized the treatment of several malignancies, including non-small cell lung cancer (NSCLC). Programmed cell death protein ligand-1 (PD-L1) expression in cancer cells is one of the inhibitory mechanisms involved in tumor immune evasion by PD-1 binding and subsequent T cell impairment [1]. Several trials have reported impressive activity of anti-PD-(L)1 monoclonal antibodies alone or in combination with chemotherapy or other immunotherapeutic drugs such as anti-CTLA-4 in patients with NSCLC [2–4]. Consequently, the FDA approved the first- or second-line use of drugs such as pembrolizumab (anti-PD-1), atezolizumab (anti-PD-L1), nivolumab (anti-PD-1), or recently cemiplimab (anti-PD-1) [5] in several tumors including lung cancer.

Significant advances have been made in the search for the ideal predictive biomarker, including the recent acceptance of tumor mutational burden (TMB) and microsatellite instability-high (MSI-H) as predictive biomarkers in the tumor-agnostic use of pembrolizumab [6]. However, PD-L1 detection by immunohistochemistry (IHC) is the FDA-approved and most commonly used predictive biomarker in these patients. Nevertheless, many patients expressing high PD-L1 did not benefit from the treatment and a considerable percentage of those with low/negative PD-L1 expression did, which might be caused by its high variability [7, 8]. Along with inter-tumor variability, patients with NSCLC show substantial intra-tumoral heterogeneity and changes in PD-L1 expression can occur after first-line treatments, hindering the accurate classification of PD-L1 status [9]. Therefore, aside from the lack of “real-time” information, a single tissue biopsy may not be able to recapitulate the exact status of the tumor microenvironment at the time of treatment that, in some cases, can be months or even years after tumor collection. Thus, there is a huge need to identify reliable predictive biomarkers for anti-PD(L)1 agents that can reflect the status of the tumor microenvironment in real-time.

PD-L1 protein expression can also be found in extracellular vesicles (EVs) [10]. These vesicles are double‐membrane structures of 20 – 2000 nm involved in intercellular communication and found in body fluids such as blood [11]. EVs are involved in the cross-talk within the tumor microenvironment and play a role in the inhibition of the anti-tumor immune response and metastasis, in particular by PD-L1 presentation [12–15] So far, only few studies have evaluated the potential of PD-L1 expression in EVs as a predicting biomarker in patients with lung cancer undergoing ICIs and they showed conflicting results that warrant further investigation [16, 17].

On the other hand, radiomics is a rapidly growing field in imaging, which can convert a patient’s imaging scans into mineable quantitative data to better understand the tumor heterogeneity and microenvironment [18]. In particular, radiomics may predict immunotherapy response and outcome in multiple cancers, including NSCLC and other advanced solid tumors [18–21]. Although combining independent predictive markers has been recommended to improve accuracy for treatment response prediction, to the best of our knowledge, only one study has attempted to combine radiomics and liquid biopsy data to predict the response to immunotherapy in patients with NSCLC [16]. Considering the limitations of that study regarding a very small cohort, using non-contrast computed tomography (CT), and most likely model overfitting, we believe that further clinical evaluation of these combined markers is needed.

There is compelling evidence that the anti-tumor immune response is a complex process regulated by the interaction between the tumor, the immune system, and multiple host factors in which EVs play a critical role. Thus, we aimed to identify and validate the predictive role of EV PD-L1 dynamics in patients with advanced/metastatic NSCLC treated with ICIs compared to the standard-of-care tissue PD-L1. Additionally, we aimed to create and evaluate the performance of a multiparametric predictive model with the inclusion of radiomics analysis in our initial cohort.

## Materials and methods

### Study design and patients

**Fig. 1:**
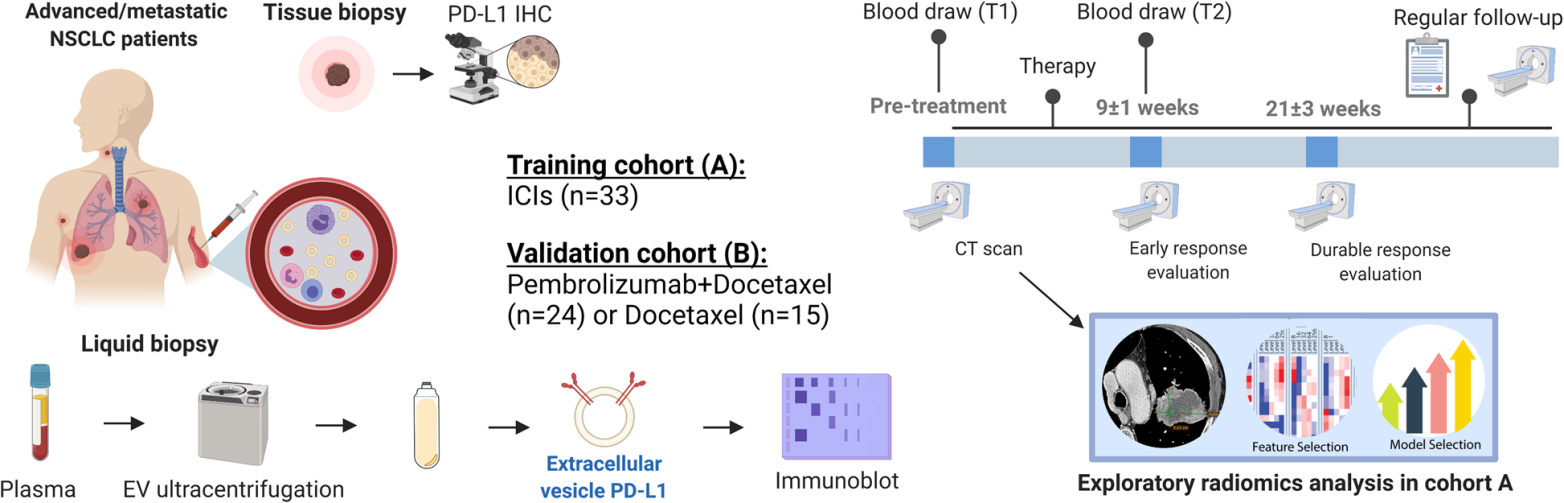
Study design: Graphical scheme of patient accrual, follow-up, and biomarker analysis [created with Biorender.com]

We conducted the retrospective analysis of blood samples and CT scan images from patients with advanced/metastatic NSCLC treated with anti-PD-1 antibodies at the Medical Oncology Unit of A.O. Papardo of Messina, Italy, between May 2018 and November 2019 with follow-up until August 2021 (Training cohort A). Then, we prospectively analyzed blood samples from patients with advanced/metastatic NSCLC enrolled in the phase 2 PROLUNG clinical trial [22], undergoing Pembrolizumab + Docetaxel or Docetaxel alone at the National Cancer Institute, Mexico, with follow-up until August 2021 (Validation cohort B) (Fig. 1). All patients provided written informed consent and the study was approved by each institutional review board. Inclusion criteria considered patients older than 18 years old at the time of diagnosis, stage IIIB or IV according to the 8th edition of the American Joint Committee on Cancer TNM manual [23], and immune-naïve patients treated with anti-PD(L)-1 (nivolumab, pembrolizumab) inhibitors in the first, second, or third line.

Histological PD-L1 expression was assessed by immunohistochemistry using SP263, 22C3, or 28–8 clones as per clinical practice in the most recent formalin-fixed paraffin-embedded tissue block from the primary tissue or metastasis and classified according to the tumor proportion score (TPS). Tumor assessment during treatment was evaluated by CT scan after 9 ± 1 weeks and at 21 ± 3 weeks of treatment according to the Response Evaluation Criteria in Solid Tumors (RECIST) version 1.1 [24]. Additionally, an independent institution review of the CT scan from the discovery cohort was conducted using RECIST v1.1 and Immune-related Response Evaluation Criteria In Solid Tumors (irRECIST) [25] for the radiomics analysis. Durable responders included those patients demonstrating complete response (CR), partial response (PR), or stable disease (SD) at the 21 ± 3 week evaluation while non-responders included those with progressive disease (PD). Progression-free survival (PFS) was defined as the time elapsed between the start of the treatment to the first radiological or clinical progression and overall survival (OS) as the time from the start of the therapy till exitus.

### Blood samples

Three mL of peripheral blood were collected into EDTA Vacutainer® tubes at baseline (T1) and at the first response evaluation (T2). Blood samples were centrifuged at 2,000 × g for 15 min and plasma was isolated and frozen at -80 °C.

### Extracellular vesicle isolation

Extracellular vesicles were isolated according to standard protocols from our group [26, 27]. Briefly, plasma aliquots were thawed and each 500 µl were diluted into 1 mL of 1X PBS and centrifuged at 3,000 × g for 20 min at 4 °C and later at 10,000 × g for 30 min at 4 °C to remove cell debris. Supernatants were recovered, diluted with 1X PBS, and centrifuged at 100,000 × g for 70 min at 4 °C into 6.5 mL, Open-Top Thickwall Polycarbonate Tubes (Beckman Coulter) in an Optima MAX Ultracentrifuge (Beckman Coulter). Finally, EVs pellets were resuspended in 1X PBS or lysed with 1X RIPA lysis buffer (Cell Signaling) and sonicated for 2 min for further analysis.

### Extracellular vesicle PD-L1 characterization

Following the last recommendations of the International Society of Extracellular Vesicles (ISEV) [28], EVs were characterized by nanoparticle tracking analysis (NTA), transmission electron microscopy (TEM), and western blot following our standardized methodology [27]. In addition, immunogold TEM characterization of PD-L1 expression in EVs was performed. EV PD-L1 expression was evaluated by immunoblot and EV PD-L1 dynamics (ΔEV PD-L1) were calculated as the expression of PD-L1 normalized against CD9 in the second time point divided by the same value in the paired baseline sample [(PD-L1/CD9) T2 / (PD-L1/CD9) T1]. A full description of the EV characterization methodology can be found in the Supplementary Methods. Increase in EV PD-L1 was defined as patients with ΔEV PD-L1 > 1 and decrease in those with ΔEV PD-L1 < 1. Patients with lower volume or quality of available plasma were excluded from the study (Cohort B: 2 patients in the Pembrolizumab + Docetaxel and 4 in the Docetaxel group).

### Radiomics imaging analysis

Radiomics analysis of target and non-target lesions was executed according to our established methodology [21]. Briefly, all lesions were segmented using 3D Slicer 4.10.1 module (Slicer 4.10.1: Summary, Highlights and Changelog—Announcements / Release Notes—3D Slicer Community) by a different color label. Additional volumes of interest (VOI) of the normal pectoralis major muscle were segmented for within-phase normalization. Ten intensity-level histogram features and 195 Gy level co-occurrence matrix (GLCM) features were extracted [29, 30]. We calculated the average, range, and angular variance of each feature for different angles, resulting in 39 rotation-invariant texture features calculated for each VOI for five gray levels. Additionally, we computed 195 volume-dependent second-order features by dividing each GLCM feature by the volume of the segmented lesions; therefore, a total of 400 radiomics features were acquired. Radiomics analysis was performed using our in-house pipeline in Matlab (version 2017b; MathWorks Inc) and Phyton Programming Language (version Phyton 3.7).

### Bioinformatics and statistical analysis

Statistical analysis and graphs were done using SPSS [SPSS Statistics for Windows, Version 22.0 (IBM Corp., Armonk NY, US), GraphPad Prism Version 8.4 (GraphPad Software Inc., San Diego CA, US) and R software (version 3.4.0, R Foundation for Statistical Computing, Vienna, Austria). Non-parametric test evaluated differences between variables. Univariate predictive models were generated with logistic regression using glmnet function from the glmnet package in R software. Regression analysis was performed with the least absolute shrinkage and selection operator (LASSO) feature selection method to find the most relevant radiomics features associated with the response [31]. Selected features were entered into eXtreme Gradient Boosting (XGBoost) to build a classification model for predicting of tumor response to immunotherapy [32]. Finally, leave-one-out cross-validation (LOOCV) was applied to assess the robustness of our models. Feature selection, model building, and receiver operating characteristics (ROC) analyses were implemented using the R packages XGBoost (version 0.6.4.1), mlr (version 2.11), and pROC (version 1.9.1). The area under the curve (AUC) was calculated for each ROC curve and sensitivity and specificity values were shown for the optimal cut-point value from each curve, resulted by selecting the value providing higher overall sensitivity & specificity (Youden’s index). Survival analyses were performed by Kaplan–Meier (log‐rank test) and Cox Proportional‐Hazards Regression with backward stepwise selection for the multivariate model. Two-tailed *p* values < 0.05 were considered statistically significant.

## Results

This study enrolled two independent cohorts of patients with advanced/metastatic NSCLC. The training cohort (A) included 33 patients undergoing ICIs with a median follow-up of 12.4 months (range 2.5 – 33.1). The validation cohort (B) enrolled 39 patients with median follow-up of 13.1 months (range 3.5 – 56.5) from which 24 received Pembrolizumab + Docetaxel and 15 Docetaxel alone. Patients characteristics are summarized in Supplementary Table S1.

### EV PD-L1 characterization

**Fig. 2:**
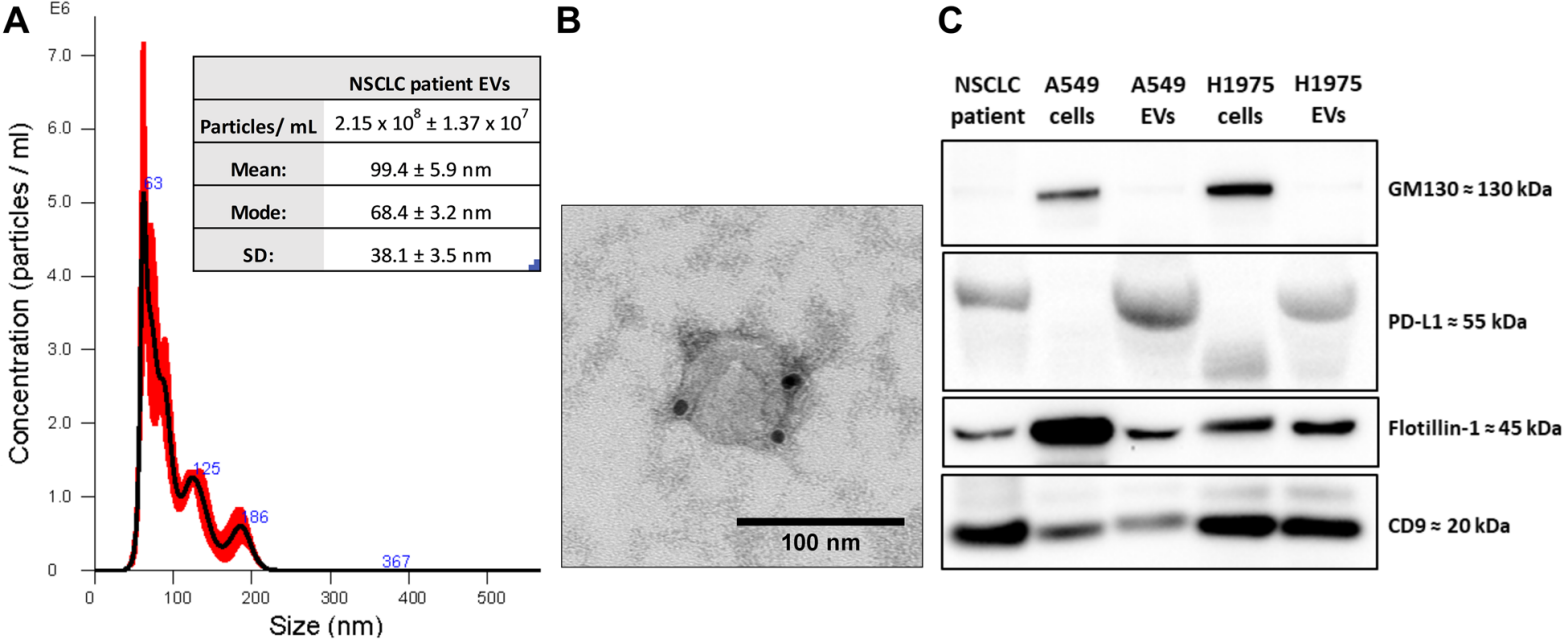
EVs characterization**:** (**A**) Nanoparticle tracking analysis (NTA) of EVs isolated from advanced NSCLC plasma samples showing a concentration of 2.15 × 10^8^ particles/mL with a mode diameter of 68.4 nm. (**B**) The immunogold transmission electron microscopy (TEM) depicted EVs of similar size with expression of PD-L1 in the membrane. (**C**) Western blot (WB) images revealed expression of PD-L1, Flotillin-1, and CD9 in the plasma EVs and lung cancer culture EVs, while low expression of GM130

Concentration, morphology, size, and specific markers in plasma EVs were analyzed to prove their nature and purity. First, the NTA showed that plasma EVs had a concentration of 2.15 × 10^8^ particles/ mL and a mean diameter of 99.4 nm (Fig. 2A). Second, the immunogold TEM characterization depicted EVs of similar size with positive PD-L1 membranous expression (Fig. 2B). Third, the western-blot revealed expression of PD-L1 and the EV markers CD9 and Flotillin-1 in EVs while absence of the non-EV marker GM130, commonly used as control for non-EV contamination (Fig. 2C). Then, PD-L1 and CD9 expression were analyzed in paired samples from each patient, calculating the ΔEV PD-L1 (Supplementary Fig. S1).

### EV PD-L1 dynamics are a better predictive biomarker for durable response to ICIs than tissue PD-L1

**Fig. 3:**
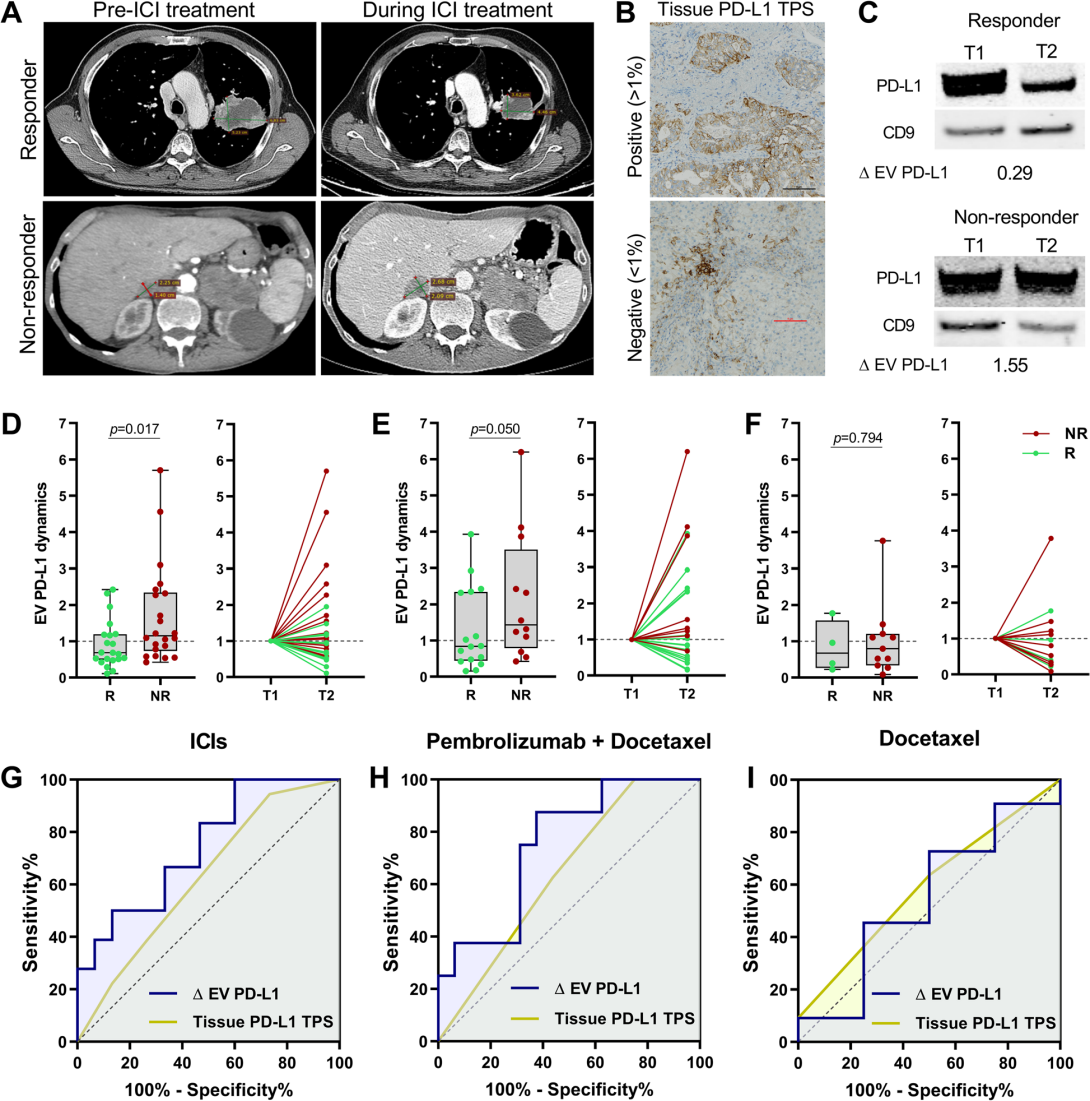
EV PD-L1 dynamics outperformed tissue PD-L1 as a predictor of ICIs response: (**A**) Representative axial section computed tomography (CT) images from a responder and a non-responder at baseline and during ICIs treatment. (**B**) Examples of immunohistochemistry micrographs of positive and negative tissue PD-L1 staining (scale bars 5 µm) and (**C**) EV PD-L1 blots from a responder with decreasing EV PD-L1 (0.29) and a non-responder showing an increase (1.55). (**D**) ICIs cohort A (*n* = 33), non-responders (NR) showed increased EV PD-L1 during treatment in comparison to responders (*p* = 0.017) (Mann–Whitney U test). (**E**) In the validation cohort, non-responders undergoing Pembrolizumab + Docetaxel (*n* = 24) showed a trend towards increased EV PD-L1 in comparison to responders (*p* = 0.050) while those treated with Docetaxel alone (*n* = 15) showed no differences (*p* = 0.794) (**F**) (Mann–Whitney U test). (**G**) As observed in the ROC curve, EV PD-L1 dynamics was a better predictor than tissue PD-L1 TPS with an AUC = 74.4% vs. 62.6% for the tissue (binary logistic regression). (**H**) This was also observed in the validation cohort of patients treated with ICIs with AUC = 75% for the EVs vs. 64.1% for the tissue. (**I**) In comparison, similar AUCs were observed in the Docetaxel treated group with 54.5% and 59.1%, respectively (binary logistic regression)

We analyzed the associations between the EV PD-L1 dynamics and the different clinical characteristics, including age, gender, line of treatment, histology, tissue PD-L1 (TPS), etc. in our three subgroups of patients with NSCLC, observing no statistical association with any of them (Supplementary Table S2). Then, we examined the potential role of this dynamic biomarker as a predictor of durable ICIs response compared to the standard-of-care biomarker, tissue PD-L1 (Representative images from responders and non-responders and the tissue and EV PD-L1 characterization are shown in Fig. 3A-C). We found an increase in EV PD-L1 during treatment in non-responders in comparison with decreasing levels in responders in our cohort A of ICIs patients (*p* = 0.017) (Fig. 3D). Similarly, in the Pembrolizumab + Docetaxel group, non-responders showed a trend towards increased EV PD-L1 in comparison to responders (*p* = 0.050) (Fig. 3E) while no differences were observed in the Docetaxel treated patients (Fig. 3F). No differences in these dynamics were found between the different treatments (Supplementary Fig. S2). Moreover, no association was found between the tissue PD-L1 expression and the durable response in any group. Thus, ΔEV PD-L1 outperformed tissue PD-L1 as a predictive factor for identifying patients with non-durable clinical benefits from ICIs. ΔEV PD-L1 showed an area-under-the-curve (AUC) of 77.3% in cohort A (Fig. 3G) and 75% in the Pembrolizumab + Docetaxel group (Fig. 3H), while the tissue PD-L1 showed only an AUC of 62.7% and 64.1%, respectively. Poor predictive values were observed for both biomarkers in the Docetaxel group (Fig. 3I). When considering the early response evaluated at the first CT scan, similar but not statistically significant differences were observed in Cohort A. However, as only one patient showed early PD in the Pembrolizumab + Docetaxel group, no significant differences were found (Supplementary Fig. S3).

**Fig. 4:**
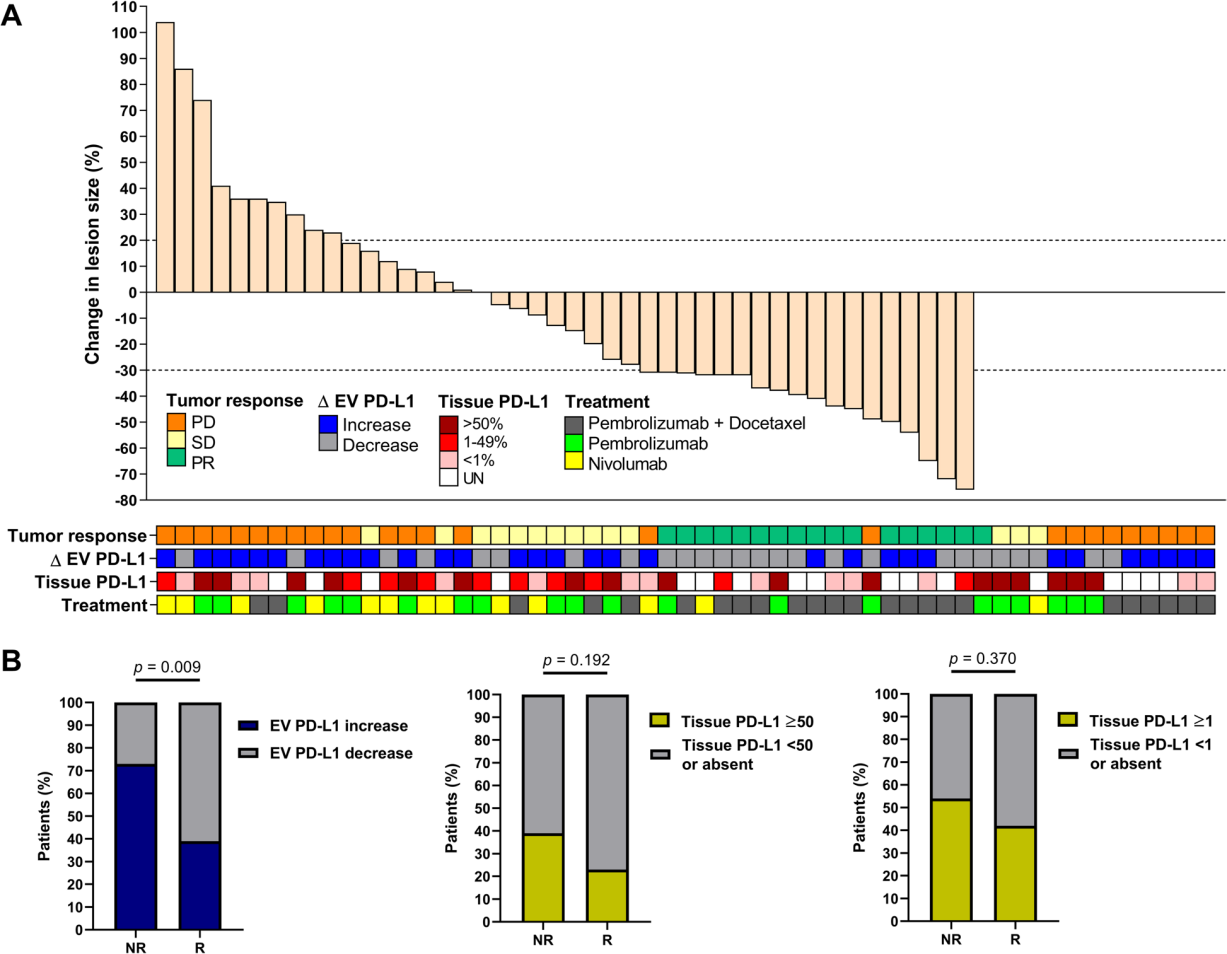
Changes in lesion size of durable response correlated with EV PD-L1 dynamics in patients undergoing ICIs. (**A**) As observed in the correlation matrix, larger increases in the tumor lesion were observed in patients with increased EV PD-L1 (*p* = 0.036) (Mann–Whitney U test) but were independent of the levels of tissue PD-L1 (*p* = 0.330) (Kruskal–Wallis test). No association was found between the tissue PD-L1 TPS and the tumor response (*p* = 0.561) (Chi-square test). (**B**) Increase in EV PD-L1 identified non-responders (*p* = 0.009), however, neither high tissue PD-L1 TPS > 50% (*p* = 0.192) or TPS > 1% (*p* = 0.370) were associated with durable response (Chi-square tests)

Furthermore, when the durable response was analyzed in all 57 patients undergoing treatment with ICIs, the dynamics of EV PD-L1 showed differences between patients with PR, SD, and PD (*p* = 0.009) since it was positively correlated with lesion size (*p* = 0.040) (Supplementary Fig. S4). Indeed, patients with increased EV PD-L1 showed an increase in lesion size (*p* = 0.036), but no association was found between the tissue PD-L1 TPS and tumor size (*p* = 0.330) or patients’ response (*p* = 0.561) (Fig. 4A). Moreover, increased EV PD-L1 identified non-responders with 73% sensitivity and 61% specificity (*p* = 0.009) (Fig. 4B). On the other hand, high tissue PD-L1 was not associated with a durable response either when considering patients with TPS ≥ 50% (*p* = 0.192) or with TPS ≥ 1% (*p* = 0.370) (Fig. 4B). Additional sub-grouped analysis of the predictive performance of EV PD-L1 across different types and lines of therapy or TPS groups are shown in Supplementary Fig. S5.

### EV PD-L1 dynamics are a predictive biomarker for survival

**Fig. 5:**
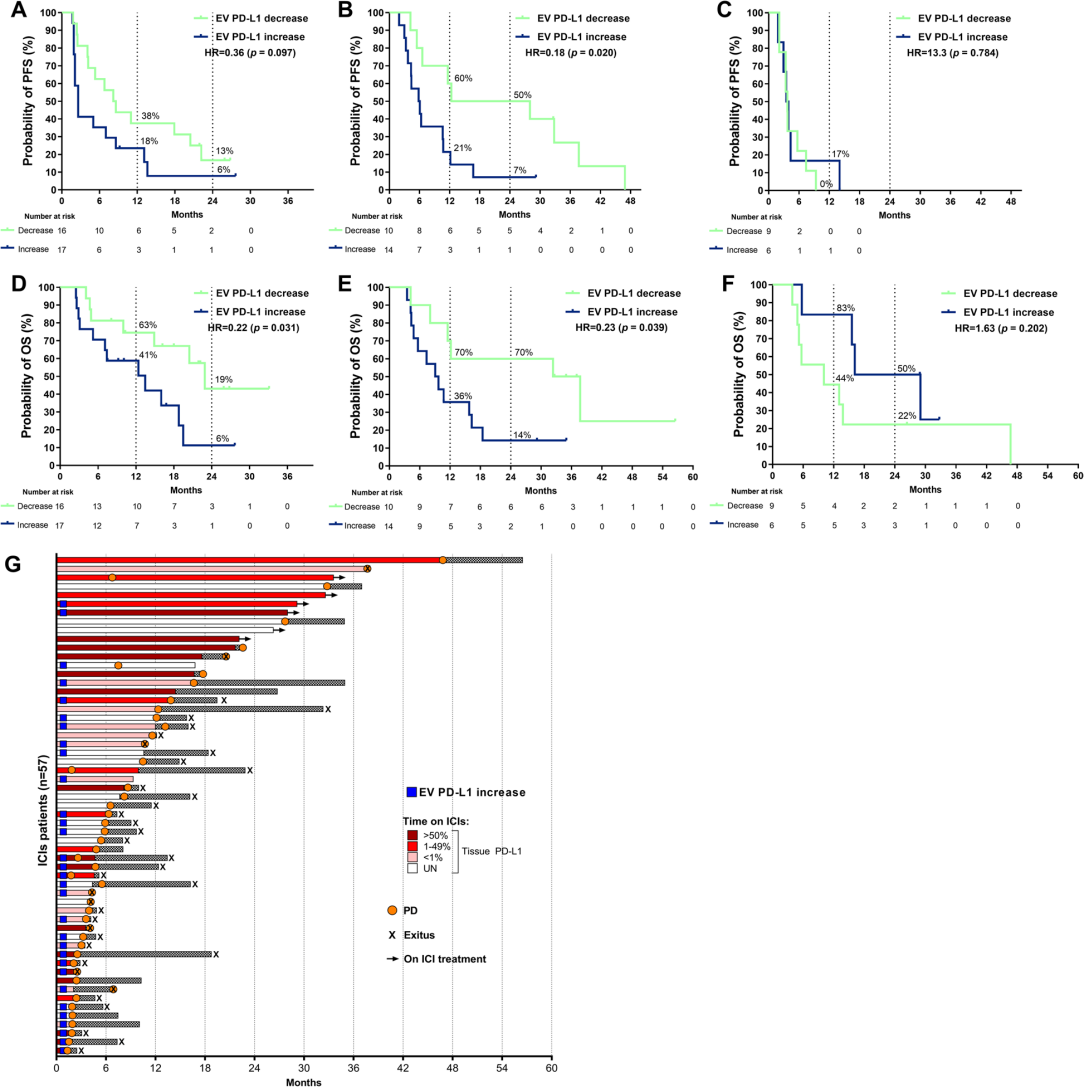
EV PD-L1 increase as a predictive biomarker for PFS and OS. (**A**) Patients with an increasing EV PD-L1 (blue) showed a trend to shorter PFS (*p* = 0.097) in the ICIs cohort and demonstrated shorter PFS in the Pembrolizumab + Docetaxel treated group (*p* = 0.020). Still, no association with PFS was observed in the Docetaxel group (*p* = 0.784) (**C**). (**D**) Longer OS was depicted in patients with EV PD-L1 increase (blue) in the ICIs cohort (*p* = 0.031) and the Pembrolizumab + Docetaxel group (*p* = 0.038) (**E**) while not in the Docetaxel control group (*p* = 0.202) (**F**) (log‐rank tests). Number of patients at risk of the event is shown every 6 months and the percentage of free of event (progression or death) patients is shown at 12 and 24 months. (**G**) In the 57 patients undergoing ICIs, an EV PD-L1 increase was observed in those with shorter PFS and OS while tissue PD-L1 was not (tissue PD-L1 TPS, dark red =  > 50%, red = 1–49%, pink < 1%, white = unknown; arrow = ongoing treatment; black & white squares bar = OS after treatment discontinuation; x = exitus (death); orange circles = progressive disease; filled dark blue rectangles = EV PD-L1 increase

During the follow-up of these cohorts, 28 (84.8%) patients progressed from Cohort A, 22 (91.7%) patients undergoing Pembrolizumab + Docetaxel, and 15 (100%) of those who underwent Docetaxel in cohort B (Supplementary Table S1). First, we analyzed the predictive value for PFS of the dynamics of EV PD-L1 in cohort A. Patients with EV PD-L1 decrease tend to experience longer PFS than those with increasing levels (Hazard ratio (HR) = 0.36; *p* = 0.097) (Fig. 5A). This was validated in patients undergoing Pembrolizumab + Docetaxel, where those with EV PD-L1 decrease showed longer PFS (HR = 0.18; *p* = 0.020) (Fig. 5B). To the contrary, no differences in PFS were observed in the Docetaxel group (HR = 13.3; *p* = 0.784) (Fig. 5C**)**. The multivariate Cox’s regression analysis for the total 57 patients receiving ICIs revealed that ΔEV PD-L1 was an independent predictive biomarker for PFS, with decreased levels associated with longer PFS (HR) = 0.45; *p* = 0.008) while tissue PD-L1 expression was not (Supplementary Table S3) (Fig. 5G).

Regarding the mortality of these patients, 19 (57.6%) patients died in cohort A. In cohort B, 18 (75%) patients died in the Pembrolizumab + Docetaxel group and 12 (80%) in the Docetaxel group (Supplementary Table S1). As previously described for the PFS, we observed that patients with decreased EV PD-L1 presented longer OS in cohort A (HR = 0.22; *p* = 0.031) (Fig. 5D). As a validation, it was also associated with longer OS in patients undergoing Pembrolizumab + Docetaxel (HR = 0.23; *p* = 0.039) (Fig. 5E) and no differences were observed in the Docetaxel group (HR = 1.63; *p* = 0.202) (Fig. 5F**)**. The multivariate Cox’s regression analysis for all 57 patients undergoing ICIs demonstrated that the decrease in EV PD-L1 was an independent predictive biomarker for longer OS (HR = 0.35; *p* = 0.004); however, the tissue PD-L1 was not (Supplementary Table S4) (Fig. 5G).

### Radiomic features complement EV PD-L1 for the prediction of the response

**Fig. 6:**
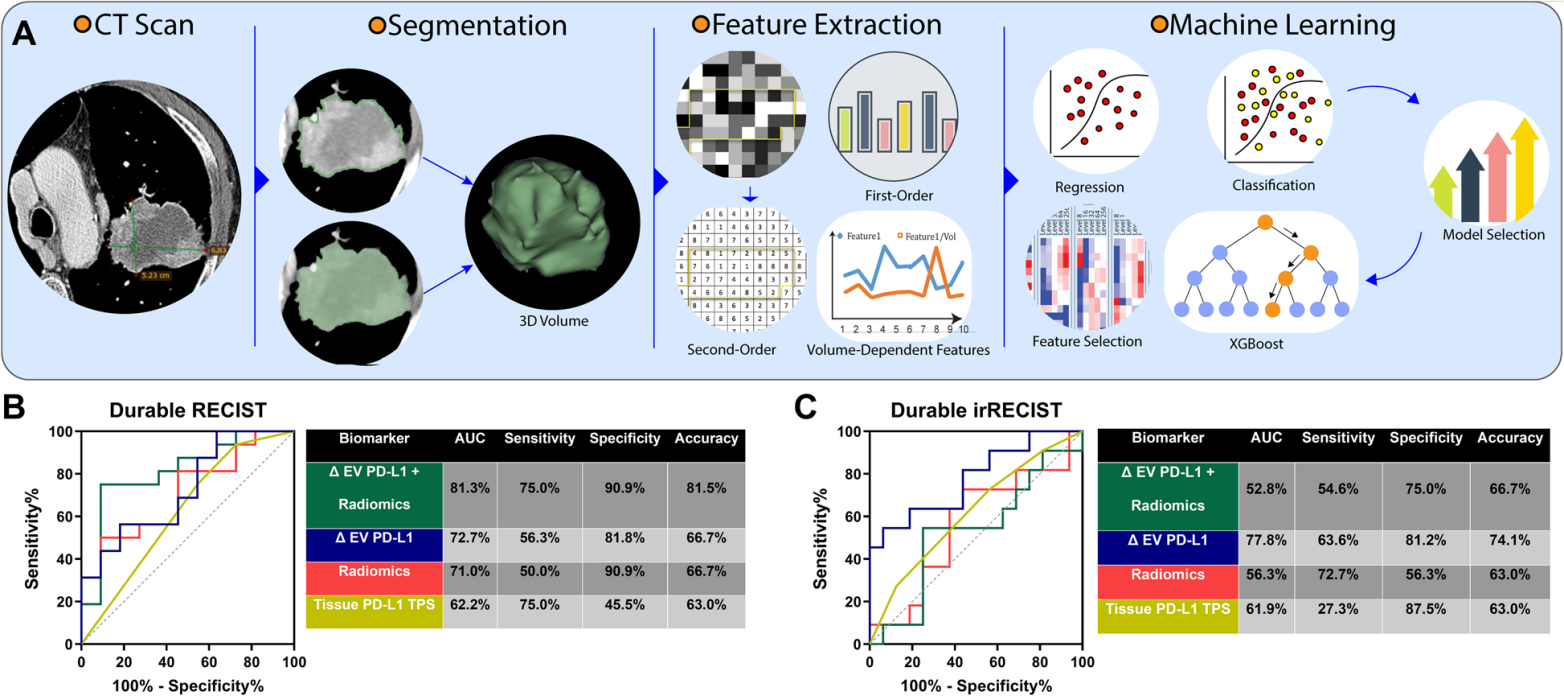
Combination of radiomics and EV PD-L1 dynamics for predicting durable response: (**A**) Characteristic pipeline for radiomic analysis including CT scan image segmentation, feature extraction, and feature and model selection by machine learning. (**B)** The introduction of the 6-features radiomic signature into the ΔEV PD-L1 predictive model for RECIST improved its performance as observed in the considerable increase of sensitivity and specificity, with an accuracy of 81.5%. (**C**) On the contrary, the best model for prediction of irRECIST only included the ΔEV PD-L1 with an accuracy of 74.1% (binary logistic regression)

Additionally, we performed an exploratory analysis of radiomics data obtained from baseline CT scans from 27 patients from our training cohort (A). Among these patients, 11 (40.7%) were classified as durable responders by RECIST and 16 (59.3%) by irRECIST, while 15 (55.6%) patients were considered early responders by RECIST and 18 (66.7%) by irRECIST, in the first CT scan. Figure 6A depicts the radiomics pipeline for feature extraction and model selection, where the most relevant features to predict RECIST durable response were selected and combined, resulting in a model of 6 LASSO features (Supplementary Table S5). We compared the predictive value of this signature with the tissue and EV PD-L1. We observed that for RECIST response, the combination of ΔEV PD-L1 and the radiomics signature was the best model, able to identify non-responders with an 81.5% accuracy. At the same time, for durable irRECIST, individual ΔEV PD-L1 was the best predictive model with 74.1% accuracy (Fig. 6B & C). Similarly, when predicting early response, the combination with radiomics also improved the predictive accuracy of the dynamics of EV PD-L1 for early RECIST response but not for irRECIST response (Supplementary Fig. S6**).** Furthermore, these six features were used to predict survival, which showed that only low TL_FLV7 was associated with worse PFS (HR = 5.52, *p* = 0.019) (Supplementary Fig. S7 & S8**)**.

## Discussion

Advanced stages of NSCLC are characterized with a suppressed immune system with reduced counts of CD8 + T-cells, responsible for the immune response against tumors [33]. Consequently, ICIs have become one of the most promising therapeutic options, revolutionizing the therapeutic landscape of these patients. Nevertheless, the efficacy of these treatments can still be primarily improved with the adequate use of reliable predictive biomarkers that could stratify which patients would benefit from them and avoid unnecessary adverse events for those who would not derive benefit. Nowadays, tissue PD-L1 is the standard-of-care for patient stratification, however, it fails at predicting the efficacy of ICIs due to several technical and biological issues associated with PD-L1 IHC [34]. Moreover, the high complexity of the immune landscape of NSCLC suggest that many markers might be involved in the response [35] and hence are needed for its prediction. In this scenario, liquid biopsy holds promise as the real-time characterization of tumors through the study of molecules found in human body fluids, able to track lung tumors evolution over time [11]. Tissue and blood TMB or dynamics of ctDNA have been proposed as potential biomarkers. However, they are still not widely used in clinical practice due to the lack of method standardization and uncertain predictive value. Indeed, recent analyses of pivotal studies, including KEYNOTE-189 [36], KEYNOTE-021 [37], or the recent results of the BFAST cohort C study [38], raised several concerns on the validity of these beforehand promising biomarkers, requiring the evaluation of other alternative circulating markers of efficacy. Therefore, we examined the predictive role of plasma EV PD-L1 expression in a retrospective cohort of patients with advanced/metastatic NSCLC undergoing treatment with ICIs and validated it in a prospective analysis of a sub-cohort from the phase 2 PROLUNG clinical trial [22]. Furthermore, in an exploratory analysis, we included radiomics data in a multiparametric predictive model in combination with the EV PD-L1.

First, we demonstrated that PD-L1 can be expressed in EVs in plasma from patients with advanced NSCLC, confirming previous findings observed in other solid tumors [39]. Then, we analyzed the dynamics of EV PD-L1 levels from baseline to 8 weeks of anti-PD-1 treatment in these patients. We observed that these dynamic changes were associated with durable responses to the treatment, since non-responders showed increased levels in comparison to a decrease in responders, even correlated with the change in lesion size. This concurs with previous studies on EV PD-L1 in patients and mice models receiving ICIs in which the increase in EV PD-L1 correlated with poor response, disease activity, or lesion size [15, 39, 40]. Similarly, a smaller study analyzed the dynamics of PD-L1 expression but focused only on exosomes from 44 patients with melanoma undergoing pembrolizumab [13]. Contrary to our results, this study described increased levels of exosomal PD-L1 in both responders and non-responders by irRECIST, observing a higher increase during the 6^th^ week of treatment in responders but equal levels at the 9^th^ week. The idea of increased levels being a predictor of better response contradicts their results and those from other studies which demonstrated that exosomal PD-L1 inhibited CD8 + T-cell function and facilitated tumor growth in in vitro and in vivo models [13, 15, 41]. This discrepancy could be caused by their use of the best response by irRECIST as classification for the clinical response, which would include as responders, patients with new metastasis or oligoprogression, or even those who shortly responded and then progressed. In addition, the difference in timepoints for second blood collection (6^th^ vs 9^th^ ± 1 week), lack of an independent validation cohort, and the analysis of exosomes and microvesicles separately, observing lower expression of PD-L1 in microvesicles than in exosomes, could explain these differences.

Moreover, we examined the potential application of EV PD-L1 in clinical practice by comparing it to the standard-of-care tissue PD-L1. The comparison between the respective predictive models showed that the dynamics of EV PD-L1 outperformed the tissue in both cohort A and B and were also able to statistically predict durable responses when analyzing all 57 ICIs patients with an 73% sensitivity and 61% specificity. Meanwhile, the tissue showed no statistically significant differences according to the response. However, the comparison in cohort B should be interpreted with precaution as tissue PD-L1 data was not available in several of these patients and they were included in the “low or absent” group.

In this context, the only similar but retrospective study in patients with NSCLC, reported a slightly increased predictive performance for tissue PD-L1 than EV PD-L1 dynamics (75% sensitivity and 57.14% specificity for EV vs. 71.43% sensitivity and 75% specificity for tissue) [16]. Nevertheless, that study only included 11 subjects in the comparison of marker performance and evaluated early response. Also, these differences could also be caused by the different methods used for EV isolation or and their use of mRNA PD-L1 expression in their analysis, contrary to our use of protein PD-L1 expression. Of particular interest, mRNA levels of PD-L1 have been demonstrated to differ from protein levels [10] as mechanisms of post-translational regulation can also alter them [42, 43]. Moreover, transmembrane PD-L1 proteins present in EVs can exert a direct inhibitory effect on CD8 + T-cells and directly inhibit immunotherapeutic monoclonal antibodies [10, 13, 14], unlike PD-L1 mRNA molecules that may need to be delivered into other cells and translated to have a biological effect. All these considerations, along with the high tumor heterogeneity in NSCLC, could explain the differences between the two studies and why dynamic protein levels of PD-L1 could be a better predictive biomarker than tissue PD-L1 and a better strategy than evaluating mRNA PD-L1expression.

Moreover, we evaluated the predictive role of the dynamics of EV PD-L1 at foreseeing survival. As observed in the independent cohort analysis and then in the multivariate analysis of all ICIs patients, a decrease in EV PD-L1 was an independent biomarker associated with patients’ longer PFS (HR = 0.45; *p* = 0.008) and OS (HR = 0.35; *p* = 0.004). In addition, neither the levels of EV PD-L1 nor the survival of ICIs patients differed between the type or line of treatment or the cohort, which increases the validity and clinical applicability of the results. As previously observed in the analysis for the prediction of response, the tissue PD-L1 was not associated with survival. These findings offer a promising new vision into the predictive value of EV PD-L1 since previous studies on melanoma [13, 39] failed to report the independent role in the multivariate analysis for survival and those in NSCLC reported no predictive value of EV PD-L1 [16], possibly due to the abovementioned differences between protein and mRNA levels on EV PD-L1 and the different protocols used. On the other hand, a recent study on 21 patients with advanced NSCLC suggested that increasing exosomal PD-L1 protein levels were associated with longer PFS and OS [17]. However, this contradicts the aforementioned role of EV and exosomal PD-L1 as an inhibitor of the immune response and of monoclonal antibodies [10, 13, 14]. Moreover, these results should be interpreted with caution due to the specific cut-off of exosomal PD-L1 selected for patient classification and the lack validation in an independent prospective cohort. In addition, as observed in their results, the use of precipitation kits for exosome isolation is not recommended for subsequent protein characterization of EVs since they are known to co-isolate abundant soluble proteins and recover less CD9 positive vesicles [28, 44], our protein of reference.

Therefore, this is the first report to show validated evidence that suggests that the dynamics of EV PD-L1 could be a reliable predictive biomarker in patients with NSCLC receiving ICIs and could potentially outperform the current standard tissue PD-L1. Moreover, EV PD-L1 lacked predictive value in chemotherapy patients, highlighting the crucial role that EVs might have in the tumor–immune interaction and their potential as specific biomarkers for ICIs. Our findings also emphasize the importance of the analysis of serial samples to track the dynamic changes in the tumors that a single biopsy might not be able to recapitulate.

Additionally, based on the promising results of radiomics being used as biomarkers for predicting response to ICIs in patients with advanced rare cancers [21], we performed an exploratory analysis of 400 radiomics features in our cohort A. Moreover, to overcome the limitations of RECIST associated with unusual imaging patterns of response, an independent radiologic evaluation of irRECIST was also performed. As a result, we observed that the combination with the radiomics signature resulted in a considerable increase in the sensitivity and specificity of the predictive model of EV PD-L1 dynamics for RECIST, which was translated into a final 81.5% predictive accuracy. In agreement with these results, one recent study reported combining radiomics with EV data increases the accuracy for predicting ICIs responses [16]. However, their reported model with 100% sensitivity and 100% specificity at predicting RECIST response likely reflects an optimistic bias caused by overfitting. The set of 11 patients was used for both model training and performance evaluation. Here, we have applied LOOCV to minimize such bias, making our results more realistic and robust. Moreover, for radiomics analysis, we used contrast-enhanced CT and assessed each patient’s entire (whole-body) tumor burden by segmentation of all target and non-target lesions, in comparison to the radiomics analysis of only primary tumors acquired from non-contrast-enhanced CT that was performed by Del Re M. et al*.* [16]. Despite these downsides, these results are consistent with our study in highlighting the possibility of combining radiomics features and EV data in predicting response to immunotherapy. On the other hand, lower accuracy was identified when including radiomics in the EV PD-L1 model for irRECIST response, probably due to the fact that those six features were selected as the optimal for predicting RECIST response.

Similarly, Mu W. et al*.* [45] analyzed pre-treatment radiomics features from positron emission tomography (PET)/CT images to predict durable response to ICIs in patients with advanced NSCLC. Their radiomics model composed by 4 CT features reported AUCs of 69% and 64% in the test and prospective test cohorts, respectively, slightly lower than the AUC of 71% found in our patients. This might be associated with the lower and non-contrast-enhanced resolution of CT images in PET/CT compared to diagnostic CTs. Also, while they only segmented primary tumors, our study included all target and non-target lesions. Moreover, they found that radiomics features were independent predictors for PFS and OS, while we found that one of our radiomic features was a biomarker of PFS but not OS. However, our results are limited regarding the low patient population and the lack of a prospective cohort. Despite these discrepancies, the study by Mu W. et al. supports our preliminary results in the sense of the potential and complementary role of radiomics to liquid biopsy biomarkers to predict immunotherapy response and survival in patients with advanced NSCLC.

Notwithstanding the high novelty and potential clinical value of EV PD-L1 as a predictor of treatment response and survival observed in our prospective validation, we recognize our study's limitations. This includes relatively small sample size, the heterogeneity of treatments in the training cohort, and the absence of tissue PD-L1 data in a high percentage of patients in the validation cohort. Therefore, we included an additionally sub-grouped analysis of the performance of EV PD-L1 as a predictor of durable response based on line and type of treatment as well as tissue PD-L1 TPS. In comparison to the whole population analysis, higher AUC was found in patients receiving Pembrolizumab and lower in those patients receiving Nivolumab (which included mostly those patients in second- and third-line treatments with lower PD-L1 TPS). This could suggest that the dynamics of EV PD-L1 is a better predictive biomarker in patients receiving Pembrolizumab than those with Nivolumab. However, based on the small population size in each of these sub-grouped analyses, no strong conclusions can be extracted from them.

In addition, despite following the latest ISEV recommendations for studies on EVs [28], we acknowledge that the isolation of EVs and the analysis of EV PD-L1 by western-blot is specialized and may be complex to apply in a clinical setting. However, the implementation of this biomarker with the development of new devices designed for an easier EV isolation and the analysis of PD-L1 would help in the translational impact of our results. This could include the development of microfluidic devices that combine on-chip ELISA detection or characterization by subsequent high-resolution flow cytometry, which could significantly reduce the time and complexity of the protocols and facilitate the translation into the clinical routine practice.

Moreover, our pilot radiomic evaluation results might be interpreted with caution due to the lack of a validation cohort. Therefore, we performed a rigorous statistical in-sample validation that could minimize the effect of this limitation. On the other hand, our results are consistent with the hypothesis that tumors are continuously evolving entities that liquid biopsy can be used to track in real-time [11]. In addition, our data highlights the importance of measuring longitudinal changes in blood biomarkers, such as circulating tumor DNA (ctDNA), which are potential predictive biomarkers for response to immunotherapy patients with advanced solid tumors [46, 47].

## Conclusion

Altogether, the results of this validation study propose that the analysis of dynamic levels of PD-L1 in EVs could be used as a predictive model to identify patients with advanced lung cancer who would derive benefit from ICIs and present better outcomes, potentially substituting or complementing the standard-of-care tissue PD-L1.

## Supplementary Information

**Additional file 1.** Supplementary materials 

## Data Availability

The datasets generated and/or analyzed during the current study are available from the corresponding author on reasonable request.

## Abbreviations

AUC: Area under the curve
CR: Complete response
CT: Computed tomography
EVs: Extracellular vesicles
GLCM: Gray level co-occurrence matrix
HR: Hazard ratio
ICIs: Immune checkpoint inhibitors
IHC: Immunohistochemistry
irRECIST: Immune-related response evaluation criteria in solid tumors
ISEV: International society of extracellular vesicles
LASSO: Least absolute shrinkage and selection operator
LOOCV: Leave-one-out cross-validation
NSCLC: Non-small cell lung cancer
NTA: Nanoparticle tracking analysis
OS: Overall survival
PD: Progressive disease
PD-L1: Programmed cell death protein ligand-1
PET: Positron emission tomography
PFS: Progression-free survival
PR: Partial response
RECIST: Response evaluation criteria in solid tumors
ROC: Receiver operating characteristics
SD: Stable disease
T1: Baseline
T2: First response evaluation
TEM: Transmission electron microscopy
TMB: Tumor mutational burden
TPS: Tumor proportion score
VOI: Volumes of interest
XGBoost: EXtreme gradient boosting
