# Supplementary material for: Extracellular vesicle PD-L1 dynamics predict durable response to immune-checkpoint inhibitors and survival in patients with non-small cell lung cancer
Source: J Exp Clin Cancer Res. 2022 Jun 2;41:186. doi: 10.1186/s13046-022-02379-1 (PMC9161571; doi:10.1186/s13046-022-02379-1)
Supplement: Supplementary file 1 — Additional file 1. Supplementary materials [file 13046_2022_2379_MOESM1_ESM.docx]

Supplementary materials for:

Extracellular vesicle PD-L1 dynamics predict durable response to immune-checkpoint inhibitors and survival in patients with non-small cell lung cancer

**Authors:** Diego de Miguel-Perez^1,2^, Alessandro Russo^2,3^, Oscar Arrieta^4^, Murat Ak^5,6^, Feliciano Barron^4^, Muthukumar Gunasekaran^2^, Priyadarshini Mamindla^6^, Luis Lara-Mejia^4^, Christine B. Peterson^7^, Mehmet E. Er^5,6^, Vishal Peddagangireddy^5^, Francesco Buemi^3^, Brandon Cooper^2^, Paolo Manca^8^, Rena G. Lapidus^2^, Ru-Ching Hsia^2^, Andres F. Cardona^9^, Aung Naing^10^, Sunjay Kaushal^2^, Fred R. Hirsch^1^, Philip C. Mack^1^, Maria Jose Serrano^11^, Vincenzo Adamo^3^, Rivka R. Colen^5,6^ & Christian Rolfo^1,2*^.

**Supplementary Materials and Methods:**

**Extracellular vesicle characterization**

**Cell culture:** A549 and H1975 lung cancer cell lines were cultured in RPMI-1640 L-Glutamine (Gibco) supplemented with 10% Fetal Bovine Serum, 100 U/ml Penicillin, and 100 ng/ml Streptomycin in a humidified incubator with 5% CO2 at 37 °C. When cells reached 80% confluency, culture media was collected and EVs were isolated as previously mentioned. Cultured cells were washed with 1X PBS and then incubated with 0.05% trypsin 0.53 mM EDTA (Corning) for 5 min at room temperature. Then, trypsin was deactivated with complete medium and cell suspensions were spun at 1,000 x g for 5 min to collect cell pellets that were washed twice in 1x PBS, spun, and then stored at −20°C until being used.

**Nanoparticle tracking analysis:** EVs were diluted in 1X PBS (1:500) and visualized in a NanoSight NS300 system (Malvern Instruments), with the acquisition of 3 movies for 30 seconds. The analysis was performed using NTA 3.1 software.

**Immunogold TEM visualization:** For fully identifying the presence of PD-L1 in our EVs samples we performed immunogold staining additionally to TEM characterization. Extracellular vesicle specimens were adsorbed onto formvar coated Nickel grids pre-treated with 0.1% BSA and then blocked with IEM incubation buffer containing 0.2% acetylated BSA, 0.1% fish gelatin in PBS, pH 7.4 for 60min. EV containing grids were then washed and incubated with the rabbit monoclonal anti-PD-L1 (Abcam, ab205921) diluted in IEM incubation buffer for overnight at 4ºC, washed five times in incubation buffer and incubated with goat anti-rabbit secondary antibody conjugated with 10 nm colloidal gold (Aurion) for two hours at room temperature.  Grids were then washed and fixed with 2% glutaraldehyde in PBS for 5 min, quenched with 50% glycine in phosphate buffer for 15 min, rinsed with water, and stained with 0.5 % uranyl acetate in water. Grids were air dried and examined in a Tecnai T12 transmission electron microscope (Thermo Scientific) at 80 keV. Images were acquired with an AMT digital camera using AMT600 software.

**Protein characterization:**  Protein concentration was quantified with the Pierce^TM^ BCA Protein Assay Kit (Thermo Fisher). Twenty µg of each sample were run in NuPAGE™ 4-12%, Bis-Tris gels (Thermo Fisher). Proteins were transferred to polyvinylidene fluoride membranes. Membranes were blocked in 5% nonfat milk, incubated with primary antibodies rabbit monoclonal anti-PD-L1 (Abcam ab205921) and mouse monoclonal anti-CD9 (Bio Legend 312102), rabbit polyclonal anti-Flotillin-1 (CST-3253S), and mouse monoclonal anti-GM130 (BD Biosciences 610822) overnight at 4°C, and later with secondary antibodies goat anti-rabbit HRP linked (CST-7074S) and horse anti-mouse HRP (CST-7076S) or with goat anti-rabbit IR-800CW (926-32211) (Green) and goat anti-mouse IR-680RD (926-68070) (Red) (Licor) linked secondary antibodies (Cell Signaling). HRP blots were developed using Immobilon chemiluminescent HRP substrate (Millipore Sigma). Protein bands intensity was quantified using ImageJ software.

**Supplementary table S1: Characteristics of the NSCLC populations.**

|  | | **ICIs (33)** | | **Pembrolizumab+Docetaxel (24)** | **Docetaxel (15)** |
| --- | --- | --- | --- | --- | --- |
| **Characteristics** | | **Number of patients (%)** | **Number of patients (%)** | | **Number of patients (%)** |
| **Gender** | Men | 21 (63.6%) | 11 (45.8%) | | 3 (21.1%) |
|  | Women | 12 (36.4%) | 13 (54.2%) | | 12 (78.9%) |
| **Age (years)** | Mean ± Standard deviation | 68.1 ± 11.3 | 54.8 ± 13.6 | | 61.3 ± 11.0 |
| **Smoking habits** | Never smoker | 5 (15.2%) | 14 (58.3%) | | 10 (66.7%) |
|  | Former smoker | 18 (54.5%) | 10 (41.7%) | | 5 (33.3%) |
|  | Current smoker | 10 (30.3%) | 0 (0%) | | 0 (0%) |
| **Histological subtype** | Non-SCC | 25 (75.8%) | 21 (87.5%) | | 14 (93.3%) |
|  | SCC | 8 (24.2%) | 3 (12.5%) | | 1 (6.7%) |
| **Stage** | IV | 32 (97.0%) | 22 (91.7%) | | 12 (80%) |
|  | IIIB | 1 (3.0%) | 2 (8.3%) | | 3 (20%) |
| **Immunotherapy treatment** | Pembrolizumab | 20 (60.6%) | 24 (100%) | | 0 (0%) |
|  | Nivolumab | 13 (39.4%) | 0 (0%) | | 0 (0%) |
| **Line** | First | 16 (48.5%) | 0 (0%) | | 0 (0%) |
|  | Second | 15 (45.5%) | 24 (100%) | | 15 (100%) |
|  | Third | 2 (6.1%) | 0 (0%) | | 0 (0%) |
| **Tissue PD-L1 (TPS)** | Negative (<1%) | 5 (15.2%) | 8 (33.3%) | | 4 (26.7%) |
|  | Low expression (1-49%) | 6 (18.2%) | 4 (16.7%) | | 4 (26.7%) |
|  | High expression (≥50%) | 17 (51.5%) | 0 (0%) | | 1 (6.6%) |
|  | Unknown | 5 (15.2%) | 12 (50%) | | 6 (40%) |
| **Early response** | Partial response | 2 (6.1%) | 8 (33.3%) | | 5 (33.3%) |
|  | Stable disease | 18 (54.5%) | 15 (62.5%) | | 7 (46.7%) |
|  | Progressive disease | 13 (39.4%) | 1 (4.2%) | | 3 (20%) |
| **Durable response** | Partial response | 4 (12.1%) | 13 (54.2%) | | 2 (13.3%) |
|  | Stable disease | 11 (33.3%) | 3 (12.5%) | | 2 (13.3%) |
|  | Progressive disease | 18 (54.5%) | 8 (33.3%) | | 11 (73.4%) |
| **Progression** | Yes | 28 (84.8%) | 22 (91.7%) | | 15 (100%) |
|  | No | 5 (15.2%) | 2 (8.3%) | | 0 (0%) |
| **PFS (months)** | Median (range) | 5.3 (1.7-27.7) | 14.0 (1.9-46.9) | | 3.7 (1.8-14.0) |
| **Death** | Yes | 19 (57.6%) | 18 (75%) | | 12 (80%) |
|  | No | 14 (42.4%) | 6 (25%) | | 3 (20%) |
| **OS (months)** | Median (range) | 12.4 (2.5-33.1) | 11.9 (3.5-56.5) | | 13.8 (3.9-46.7) |

**Supplementary table S2: Association between clinical characteristics and EV PD-L1 dynamics.**

|  | | **EV PD-L1 dynamics** | | | | | | | |
| --- | --- | --- | --- | --- | --- | --- | --- | --- | --- |
|  | | **ICIs** | | **Pembrolizumab + Docetaxel** | | | **Docetaxel** | | |
| **Clinical characteristics** | | Median (95% CI) | *p*-value | Median (95% CI) | *p*-value | median (95% CI) | | *p*-value |  |
| **Gender** | Men | 1.05 (0.65-1.48) | 0.765 | 1.24 (0.69-2.92) | 0.750 | 0.80 (0.52-3.76) | | 0.470 |  |
|  | Women | 1.03 (0.52-1.55) |  | 0.85 (0.43-3.86) |  | 0.74 (0.27-1.20) | |  |  |
| **Age (years)** | < 65 | 1.31 (0.48-5.70) | 0.529 | 1.24 (0.54-2.92) | 0.271 | 0.52 (0.27-1.47) | | 0.724 |  |
|  | ≥ 65 | 1.05 (0.69-1.20) |  | 1.02 (0.15-1.56) |  | 1.03 (0.09-3.76) | |  |  |
| **Smoking habits** | Never smoker | 1.20 (0.52-1.71) | 0.645 | 0.84 (0.43-2.92) | 0.349 | 0.66 (0.33-1.20) | | 0.903 |  |
|  | Former smoker | 0.94 (0.55-1.22) |  | 1.22 (0.69-4.12) |  | 0.96 (0.09-3.76) | |  |  |
|  | Current smoker | 1.07 (0.58-3.10) |  | x |  | x | |  |  |
| **Histological subtype** | Non-SCC | 0.96 (0.65-1.48) | 0.644 | 1.10 (0.54-2.35) | 0.631 | 0.88 (0.33-1.47) | | 0.247 |  |
|  | SCC | 1.07 (0.11-4.56) |  | 1.56 (0.69-2.92) |  | 0.27 (NA-NA) | |  |  |
| **Stage** | IV | 1.00 (0.65-1.22) | 0.401 | 1.11 (0.54-2.35) | 0.676 | 0.66 (0.33-1.47) | | 0.665 |  |
|  | IIIB | 1.48 (UN-UN) |  | 2.31 (0.69-3.93) |  | 0.96 (0.22-1.10) | |  |  |
| **Immunotherapy treatment** | Nivolumab | 1.05 (0.58-1.22) | 0.768 | x | x | x | | x |  |
|  | Pembrolizumab | 1.03 (0.59-1.55) |  | 1.11 (0.68-2.35) |  | x | |  |  |
| **Line** | First | 1.01 (0.55-1.71) | 0.863 | x | x | x | | x |  |
|  | Second | 1.05 (0.69-1.22) |  | 1.11 (0.68-2.35) |  | 0.80 0.33-1.20) | |  |  |
|  | Third | 0.86 (0.52-1.20) |  | x |  | x | |  |  |
| **Tissue PD-L1 (TPS)** | <1% | 1.18 (0.58-3.10) | 0.601 | 1.06 (0.15-3.86) | 0.579 | 0.66 (0.22-3.76) | | 0.439 |  |
|  | 1-49% | 1.00 (0.48-2.58) |  | 1.63 (0.18-3.92) |  | 1.11 (0.39-1.20) | |  |  |
|  | ≥50% | 0.96 (0.59-1.55) |  | x |  | 1.47 (NA-NA) | |  |  |
|  | Unknown | 0.69 (0.29-1.22) |  | 1.28 (0.69-2.92) |  | 0.43 (0.09-1.77) | |  |  |

UN: Unknown, median value is not available as there is only 1 patient present per class (Mann-Whitney U and Kruskal–Wallis tests).

**Supplementary Table S3: Univariate and multivariate analysis for PFS**

| **PFS** | | **Cohort A+B (ICIs) (n=57)** | | | | | **Cohort B (Docetaxel) (n=15)** | | | | |  |
| --- | --- | --- | --- | --- | --- | --- | --- | --- | --- | --- | --- | --- |
|  |  | **Univariate analysis** | | | **Multivariate analysis** | | **Univariate analysis** | | **Multivariate analysis** | | |  |
| **Variable** | | | **HR (95% CI)** | ***p*** | **HR (95% CI)** | ***p*** | **HR (95% CI)** | ***p*** | | **HR (95% CI)** | ***p*** | |
| **Gender** | Women | 1.0 (Reference) | |  |  |  | 1.0 (Reference) |  | | 1.0 (Reference) |  | |
|  | Men | 1.14 (0.65-2.00) | | 0.653 |  |  | 5.45 (1.06-28.06) | **0.042** | | 5.45 (1.06-28.06) | **0.042** | |
| **Age** | < 65 | 1.0 (Reference) | |  |  |  | 1.0 (Reference) |  | |  |  | |
|  | ≥ 65 | 1.14 (0.64-2.00) | | 0.660 |  |  | 1.69 (0.54-5.28) | 0.369 | |  |  | |
| **Ever smoker** | No | 1.0 (Reference) | |  |  |  | 1.0 (Reference) |  | |  |  | |
|  | Yes | 1.37 (0.75-2.52) | | 0.305 |  |  | 0.32 (0.08-1.20) | 0.090 | |  |  | |
| **Histology** | SCC | 1.0 (Reference) | |  |  |  | 1.0 (Reference) |  | |  |  | |
|  | Non-SCC | 0.93 (0.45-1.93) | | 0.838 |  |  | 0.43 (0.05-3.68) | 0.440 | |  |  | |
| **Stage** | III | 1.0 (Reference) | |  |  |  | 1.0 (Reference) |  | |  |  | |
|  | IV | 4.23 (0.58-30.82) | | 0.155 |  |  | 1.69 (0.46-6.20) | 0.428 | |  |  | |
| **Cohort** | Cohort A (ICIs) | 1.0 (Reference) | |  |  |  |  |  | |  |  | |
|  | Cohort B (ICI+ChT) | 0.68 (0.38-1.24) | | 0.208 |  |  |  |  | |  |  | |
| **Immunotherapy** | Nivolumab | 1.0 (Reference) | |  |  |  |  |  | |  |  | |
|  | Pembrolizumab | 0.76 (0.38-1.50) | | 0.426 |  |  |  |  | |  |  | |
| **Line** | First | 1.0 (Reference) | | 0.790 |  |  |  |  | |  |  | |
|  | Second | 0.81 (0.42-1.55) | | 0.518 |  |  | 1.0 (Reference) |  | |  |  | |
|  | Third | 1.01 (0.22-4.58) | | 0.987 |  |  |  |  | |  |  | |
| **Tissue PD-L1 (TPS)** | <1% | 1.0 (Reference) | | 0.752 |  |  | 1.0 (Reference) | 1.000 | |  |  | |
|  | 1-49% | 0.69 (0.27-1.78) | | 0.446 |  |  | 1.00 (0.24-4.11) | 1.000 | |  |  | |
|  | ≥50% | 1.11 (0.50-2.44) | | 0.799 |  |  | 1.00 (0.00-2.7x10^4^) | 1.000 | |  |  | |
|  | Unknown | 1.11 (0.52-2.37) | | 0.789 |  |  | 1.00 (0.27-3.77) | 1.000 | |  |  | |
| Δ **EV PD-L1** | Increase (>1) | 1.0 (Reference) | |  | 1.0 (Reference) |  | 1.0 (Reference) |  | |  |  | |
|  | Decrease (<1) | 0.45 (0.25-0.81) | | **0.008** | 0.45 (0.25-0.81) | **0.008** | 1.16 (0.39-3.50) | 0.787 | |  |  | |
| **Cohort A (n=27)** |  |  | |  |  |  |  |  | |  |  | |
| **Radiomics TL_FV7** | High | 1.0 (Reference) | |  |  |  |  |  | |  |  | |
|  | Low | 2.95 (1.13-7.68) | | **0.027** |  |  |  |  | |  |  | |

Cox Proportional‐Hazards Regression

**Supplementary Table S4: Univariate and multivariate analysis for OS**

| **OS** | | **Cohort A+B (ICIs) (n=57)** | | | | **Cohort B (Docetaxel)** | | | |
| --- | --- | --- | --- | --- | --- | --- | --- | --- | --- |
|  |  | **Univariate analysis** | | **Multivariate analysis** | | **Univariate analysis** | | **Multivariate analysis** | |
| **Variable** | | **HR (95% CI)** | ***p*** | **HR (95% CI)** | ***p*** | **HR (95% CI)** | ***p*** | **HR (95% CI)** | ***p*** |
| **Gender** | Women | 1.0 (Reference) |  |  |  | 1.0 (Reference) |  | 1.0 (Reference) |  |
|  | Men | 1.39 (0.72-2.68) | 0.330 |  |  | 10.34 (1.68-63.84) | **0.012** | 10.34 (1.68-63.84) | **0.012** |
| **Age** | < 65 | 1.0 (Reference) |  |  |  | 1.0 (Reference) |  |  |  |
|  | ≥ 65 | 1.14 (0.59-2.18) | 0.703 |  |  | 1.46 (0.42-5.09) | 0.554 |  |  |
| **Ever smoker** | No | 1.0 (Reference) |  |  |  | 1.0 (Reference) |  |  |  |
|  | Yes | 1.48 (0.74-2.96) | 0.270 |  |  | 1.35 (0.38-4.86) | 0.644 |  |  |
| **Histology** | SCC | 1.0 (Reference) |  |  |  | 1.0 (Reference) |  |  |  |
|  | Non-SCC | 0.71 (0.32-1.56) | 0.389 |  |  | 26.9 (0.01-7.6 x10^4^ | 0.416 |  |  |
| **Stage** | III | 1.0 (Reference) |  |  |  | 1.0 (Reference) |  |  |  |
|  | IV | 2.48 (0.34-18.18) | 0.373 |  |  | 2.82 (0.36-22.35) | 0.327 |  |  |
| **Cohort** | Cohort A (ICIs) | 1.0 (Reference) |  |  |  |  |  |  |  |
|  | Cohort B (ICI+ChT) | 1.09 (0.56-2.11) | 0.799 |  |  |  |  |  |  |
| **ICIs** | Nivolumab | 1.0 (Reference) |  |  |  |  |  |  |  |
|  | Pembrolizumab | 1.14 (0.47-2.77) | 0.775 |  |  |  |  |  |  |
| **Line** | First | 1.0 (Reference) | 0.983 |  |  |  |  |  |  |
|  | Second | 0.94 (0.46-1.92) | 0.856 |  |  | 1.0 (Reference) |  |  |  |
|  | Third | 0.00 (0.00-NA) | 0.981 |  |  |  |  |  |  |
| **Tissue PD-L1 (TPS)** | <1% | 1.0 (Reference) | 0.702 |  |  | 1.0 (Reference) | 0.549 |  |  |
|  | 1-49% | 0.52 (0.18-1.53) | 0.235 |  |  | 0.29 (0.05-1.77) | 0.178 |  |  |
|  | ≥50% | 0.79 (0.33-1.90) | 0.602 |  |  | 0.39 (0.04-4.17) | 0.439 |  |  |
|  | Unknown | 0.79 (0.33-1.89) | 0.599 |  |  | 0.73 (0.17-3.18) | 0.676 |  |  |
| Δ **EV PD-L1** | Increase (>1) | 1.0 (Reference) |  | 1.0 (Reference) |  | 1.0 (Reference) |  |  |  |
|  | Decrease (<1) | 0.35 (0.17-0.72) | **0.004** | 0.35 (0.17-0.72) | **0.004** | 2.22 (0.63-7.78) | 0.212 |  |  |
| **Cohort A (n=27)** |  |  |  |  |  |  |  |  |  |
| **Radiomics TL_FV7** | High | 1.0 (Reference) |  |  |  |  |  |  |  |
|  | Low | 1.97 (0.69-5.62) | 0.204 |  |  |  |  |  |  |

Cox Proportional‐Hazards Regression

| **Radiomic features** | | | |
| --- | --- | --- | --- |
| **Feature** | **Lesion Type** | **Level** | **Feature Name** |
| NTL_FV26 | Non-Target | 8 | Range of Information Measure Of Correlation 2 |
| NTL_FO3 | Non-Target | - | Mean of First order |
| TL_F101 | Target | 32 | Range of Difference Variance |
| TL_FV129 | Target | 64 | Average of Information Measure Of Correlation |
| TL_FV7 | Target | 8 | Average of Sum Variance |
| NTL_FO7 | Non-Target | - | 95% Percentile of First Order |

**Supplementary Table S5: Radiomic features selected**

**Supplementary figures:**


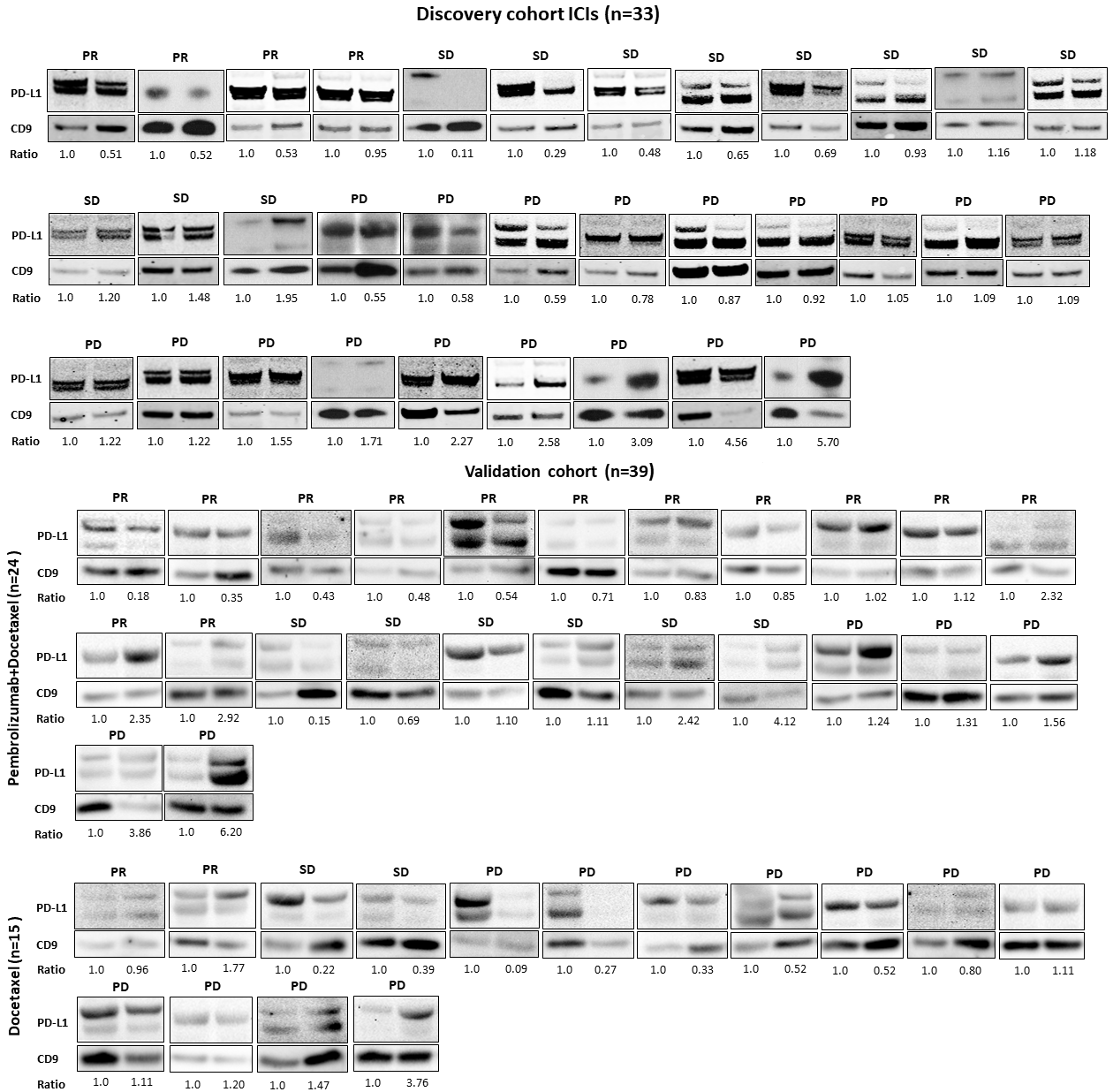


**Supplementary Fig. S1: Immunoblots for EV PD-L1 characterization in NSCLC patients.** PD-L1 and CD9 blots from samples obtained at baseline (T1) and first response evaluation (T2) in the discovery and the validation cohorts showing the EV PD-L1 value in each patient. EV PD-L1 dynamics were calculated as the EV PD-L1 value at the T2 normalized against the value at T1.


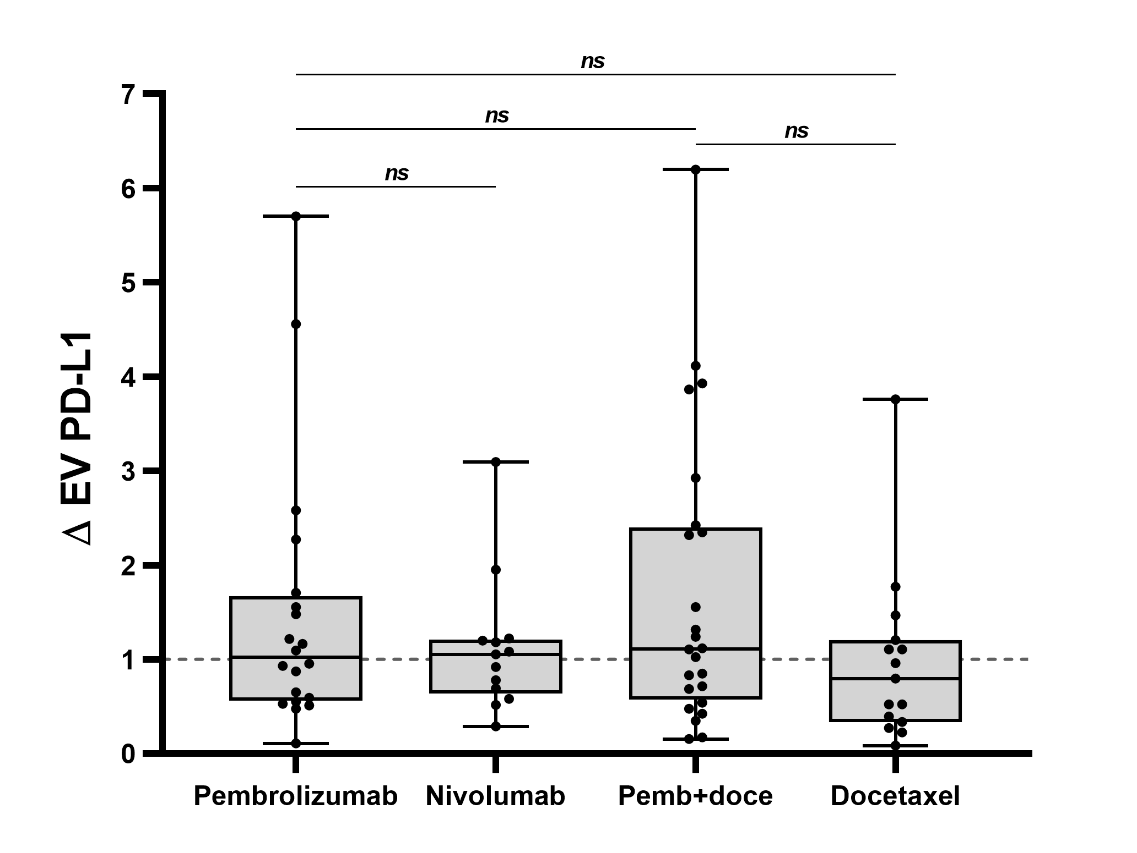


**Supplementary Fig. S2: ΔEV PD-L1 expression along the different treatments.** No differences in the ΔEV PD-L1 were observed between the different treatments received during this study (Pemb+doce: Pembrolizumab+Docetaxel) (Mann-Whitney U & Kruskal–Wallis test).


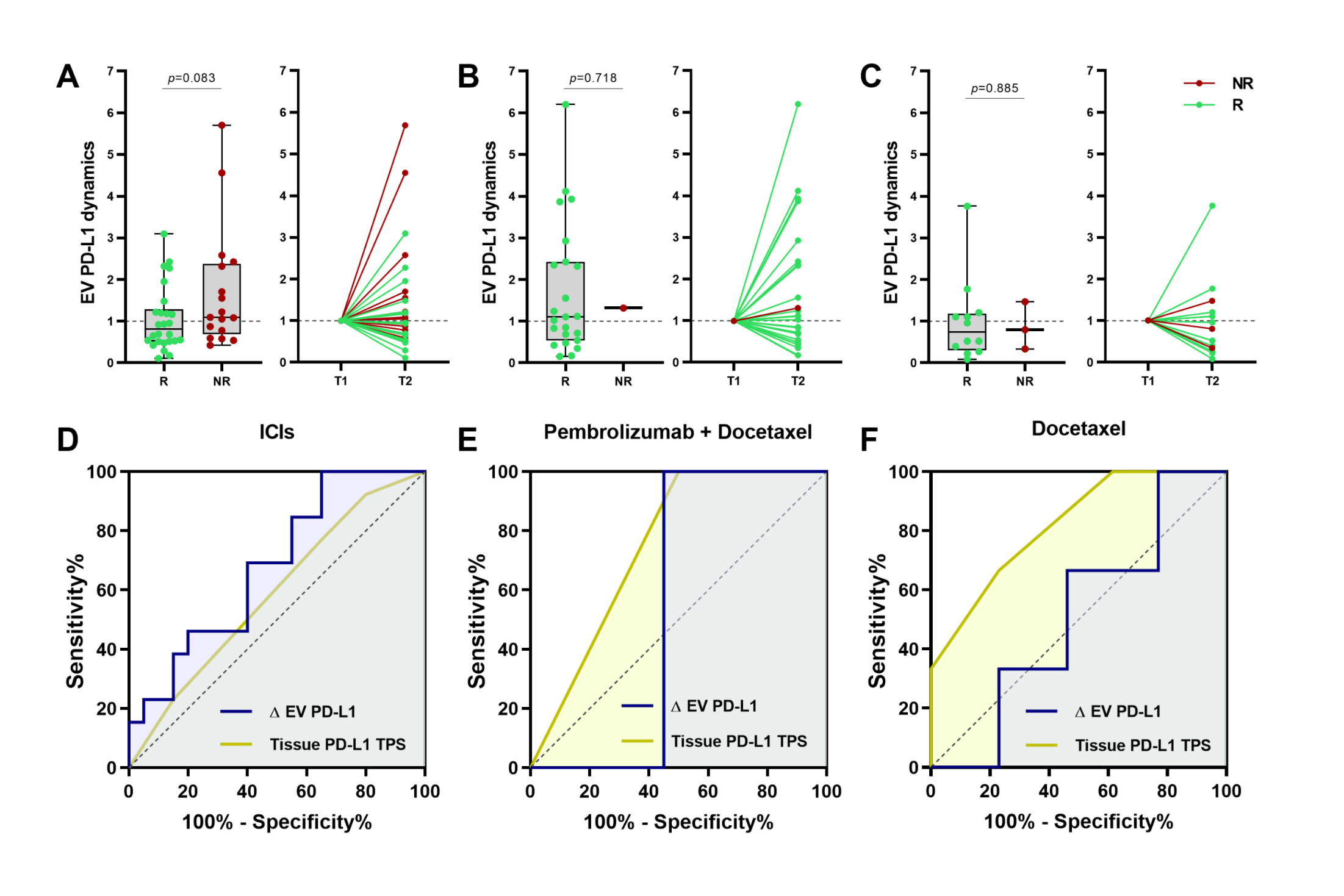


**Supplementary Fig. S3: EV PD-L1 dynamics as predictor of early ICIs response:** (A) In the ICIs cohort A, non-responders (NR) showed a trend to increased EV PD-L1 in comparison to EV PD-L1 decrease in responders. (B) In the validation cohort of patients undergoing Pembrolizumab + Docetaxel, only one patient progressed and no differences were found between responders and non-responders, similar to what was observed in docetaxel treated patients with only 3 non-responders (C) (Mann-Whitney U tests). (D) ΔEV PD-L1 was a better predictor of early response in ICIs patients from cohort A, with an AUC of 68.1% and tissue PD-L1 only showing an AUC of 58.7%. (E) In Pembrolizumab+Docetaxel patients, AUC=55.0% was observed for the EV vs. 75.0% for the tissue. (F) In Docetaxel patients, ΔEV PD-L1 presented an AUC of 51.3% vs. 82.0% for the tissue (binary logistic regressions).


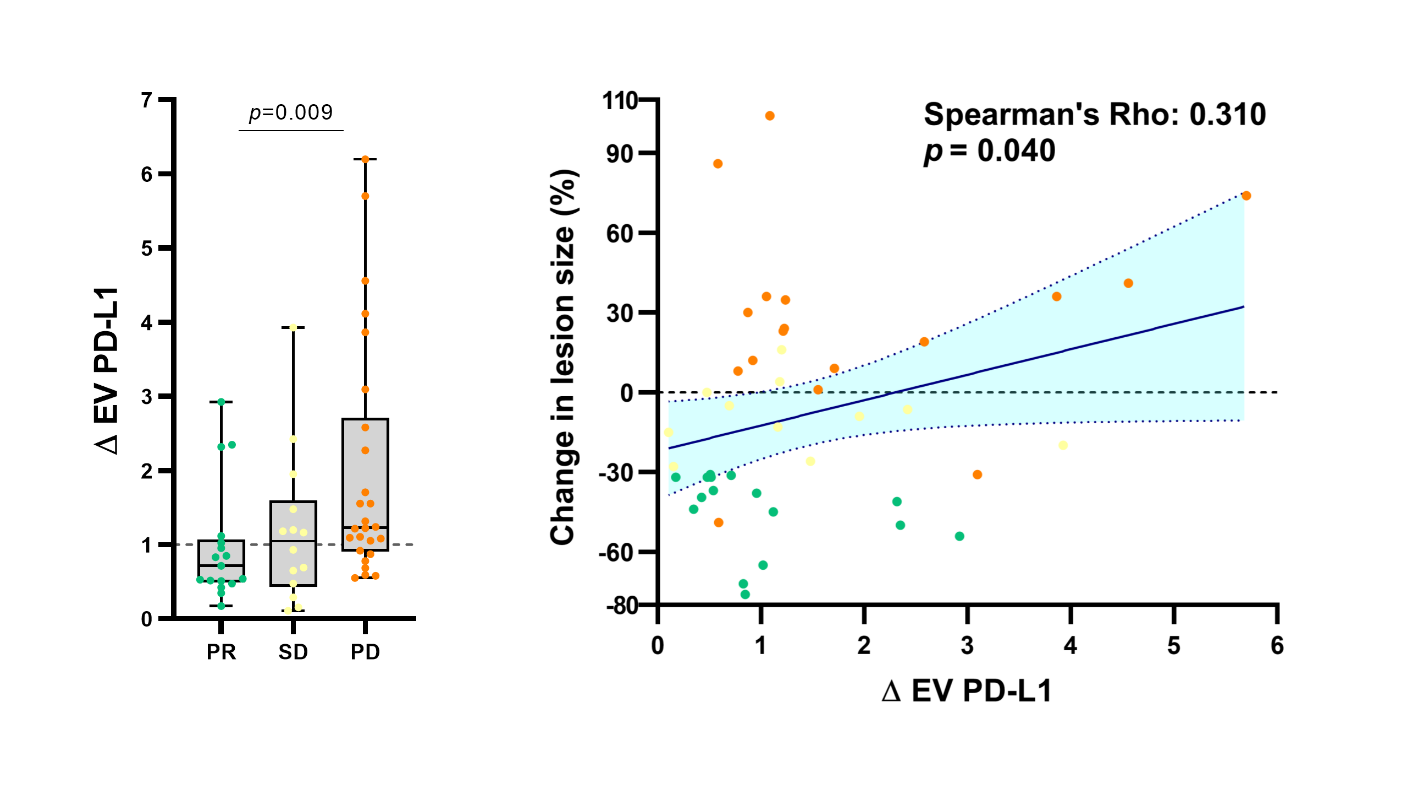


**Supplementary Fig. S4: EV PD-L1 dynamics and durable tumor response:** ΔEV PD-L1 identified PR, SD, or PD durable response in the total of 57 patients undergoing ICIs (p=0.009) (Kruskal–Wallis test) as it correlated with the change in lesion size (*p*=0.040) (Spearman’s rank correlation).


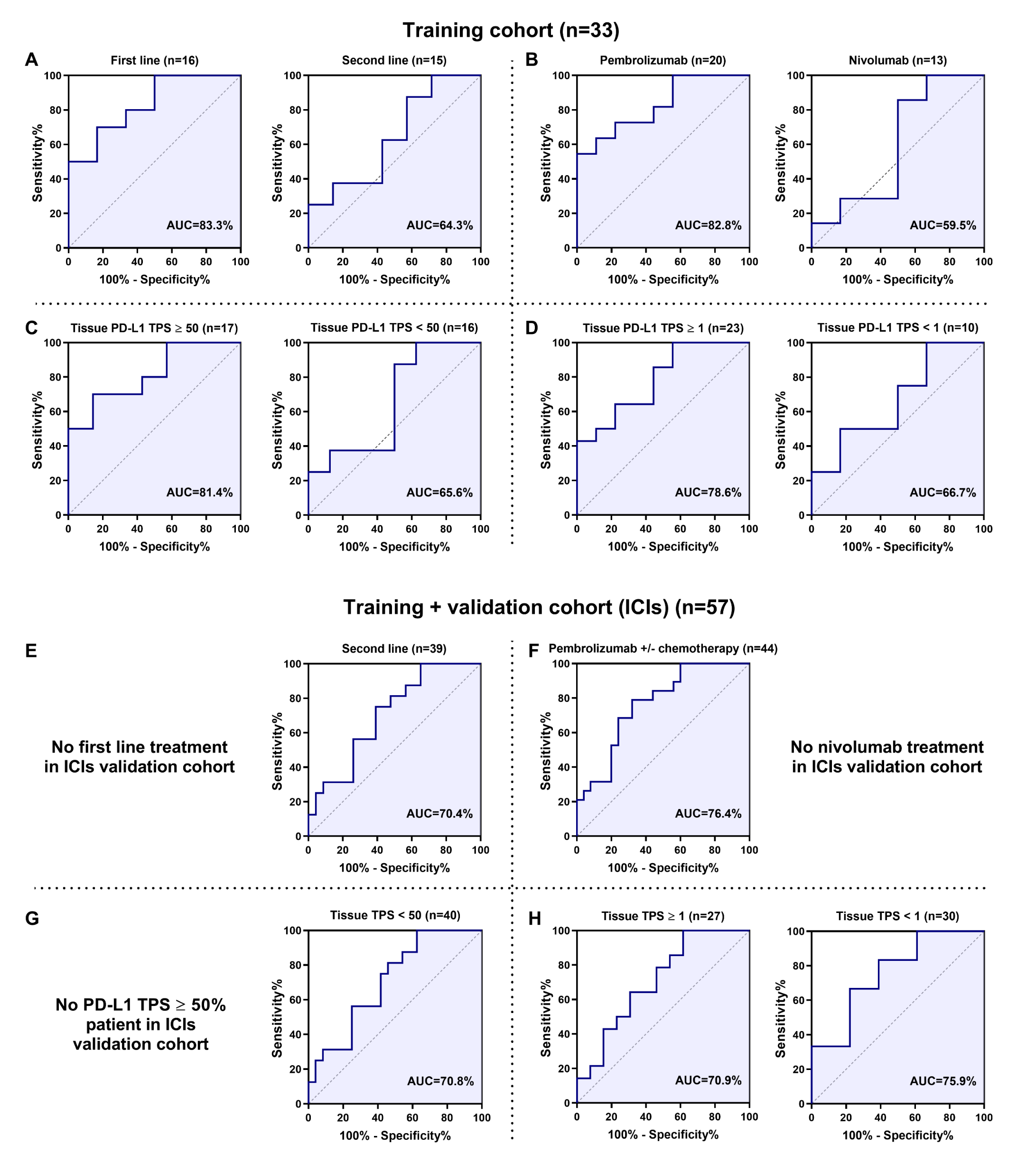


**Supplementary Fig. S5: EV PD-L1 dynamics as predictor of durable ICIs response across treatment lines, types, and tissue PD-L1 TPS:** In the training cohort, the ROC show the performance of EV PD-L1 dynamics based on the line of treatment (A) (note that only 2 patients received ICIs in third line and no graph can be created), the type of treatment (B), the positivity of the tissue PD-L1 expression based on a TPS ≥50% cutoff (C) or a TPS ≥1% cutoff (D). Similarly, when the validation cohort of 24 patients receiving second line Pembrolizumab+chemotherapy are included in the analysis, the ROC show the performance of EV PD-L1 dynamics based on the line of treatment (E), the type of treatment (F), the positivity of the tissue PD-L1 expression based on a TPS ≥50% cutoff (G) or a TPS ≥1% cutoff (H) (binary logistic regressions).

**
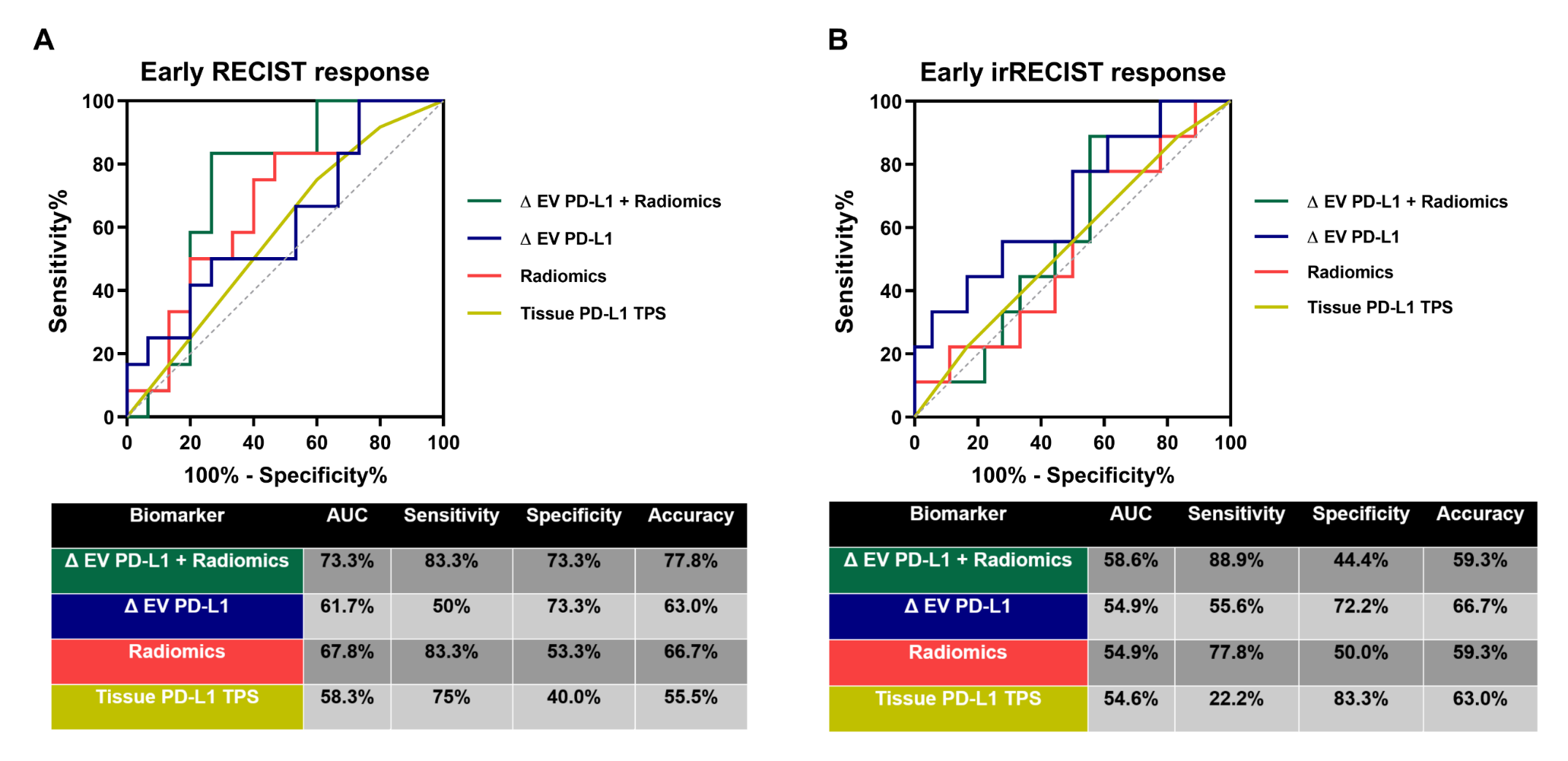
Supplementary Fig. S6: Combination of radiomics and EV PD-L1 dynamics for predicting early response:** (A) Predictive models of dynamics of EV PD-L1, radiomics, or tissue PD-L1 TPS to predict early RECIST response in patients undergoing ICIs (n=27). (B) Predictive models of dynamics of EV PD-L1, radiomics, or tissue PD-L1 TPS to predict early irRECIST response in patients undergoing ICIs (n=27) (binary logistic regressions).


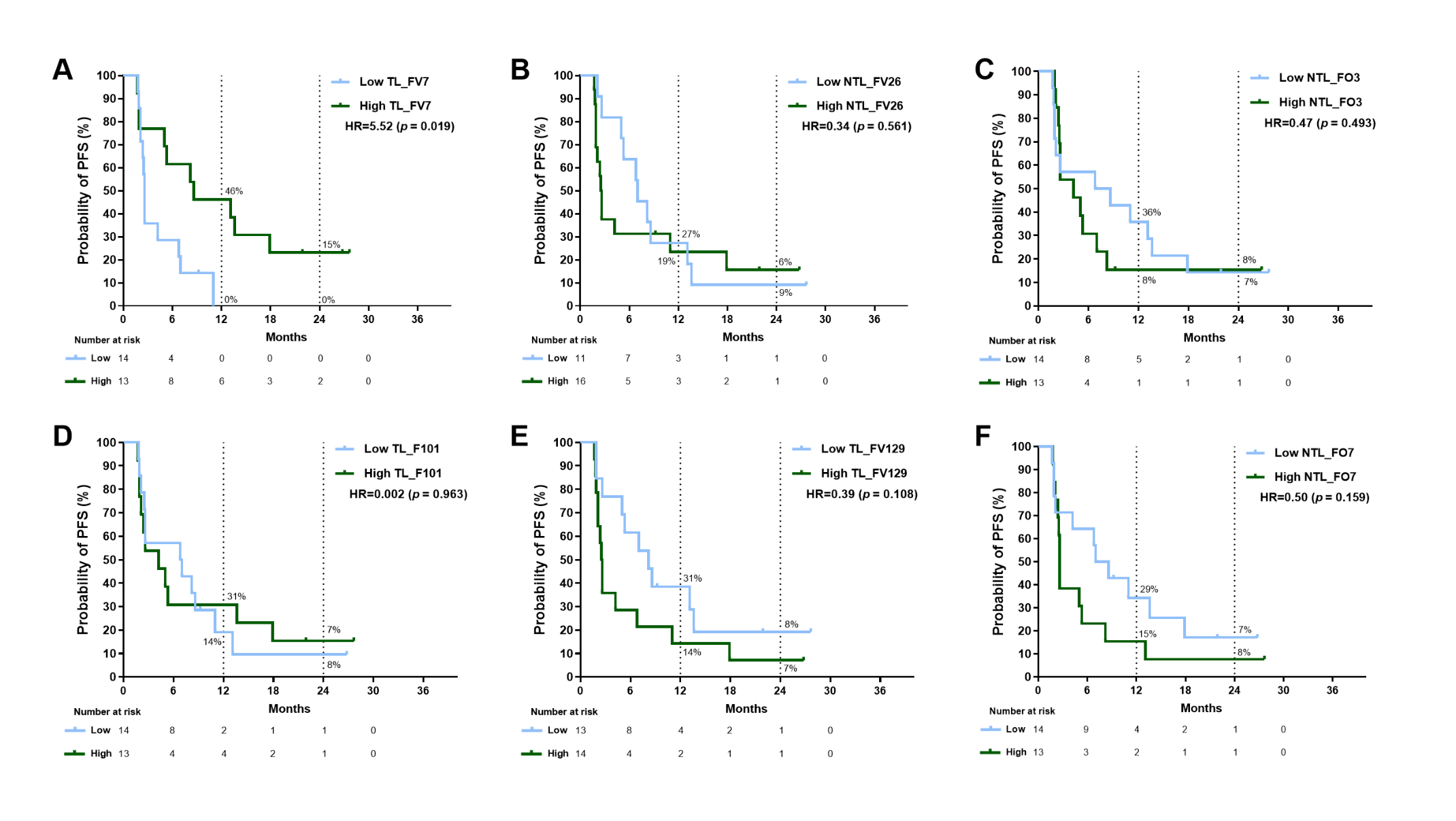


**Supplementary Figure S7: PFS according to the levels of 6 baseline radiomic features in 27 ICIs patients:** (A) Patients with high TL_FV7 showed longer PFS (p=0.019). NTL_FV26 (B), NTL_FO3 (C), TL_F101 (D), TL_FV129 (E), and NTL_FO7 (F) were not statistically associated to the PFS of these patients (log‐rank tests).


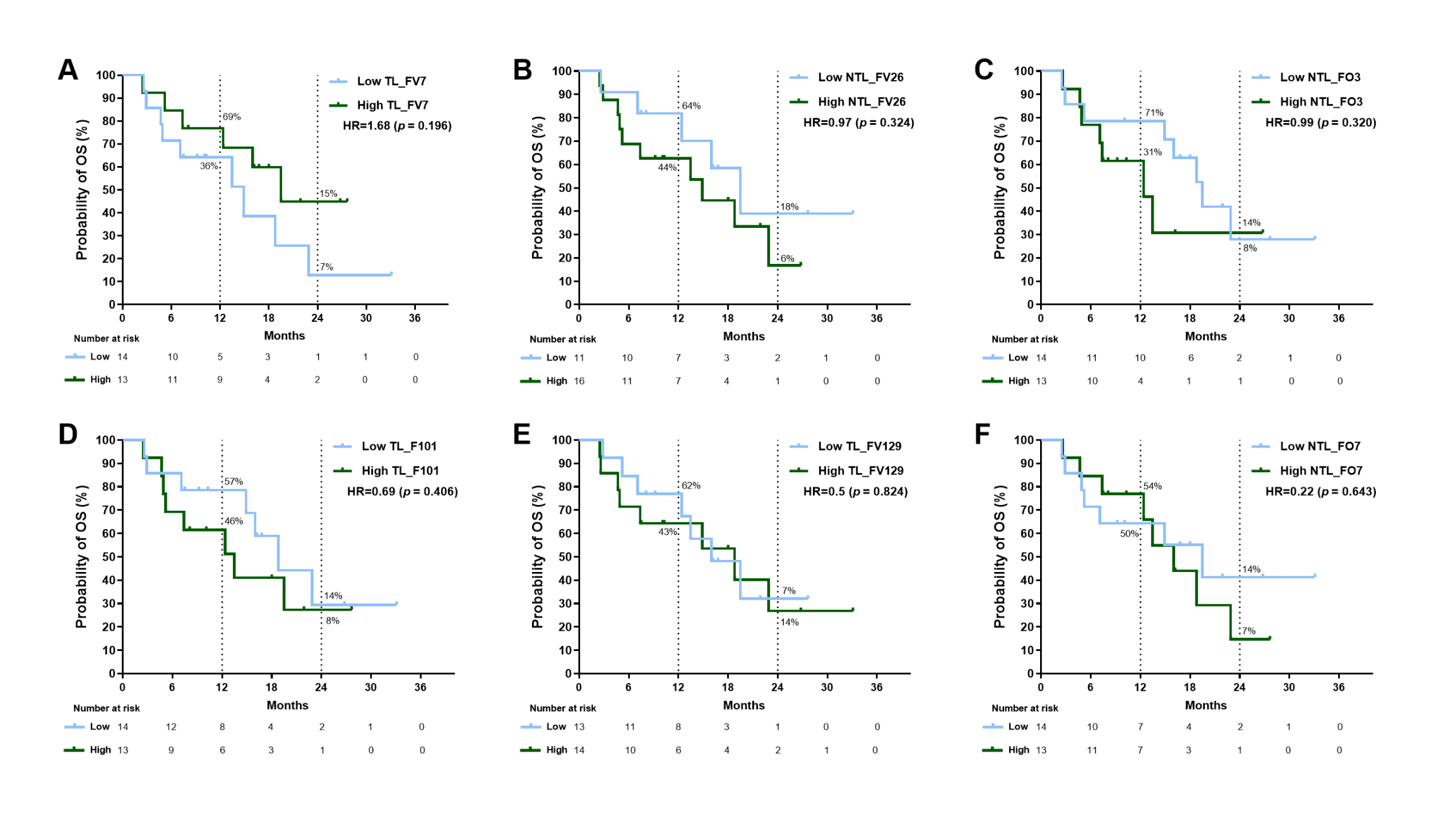


**Supplementary Figure S8: OS according to the levels of 6 baseline radiomic features in 27 ICIs patients:** (A) TL_FV7, NTL_FV26 (B), NTL_FO3 (C), TL_F101 (D), TL_FV129 (E), and NTL_FO7 (F) baseline radiomics features showed no association with the OS of these patients (log‐rank tests).
